# CaREM1.4 interacts with CaRIN4 to regulate *Ralstonia solanacearum* tolerance by triggering cell death in pepper

**DOI:** 10.1093/hr/uhad053

**Published:** 2023-03-28

**Authors:** Yanqin Zhang, Shuangyuan Guo, Feng Zhang, Pengfei Gan, Min Li, Cong Wang, Huankun Li, Gang Gao, Xiaojie Wang, Zhensheng Kang, Xinmei Zhang

**Affiliations:** College of Plant Protection, Northwest A&F University, Yangling 712100, Shaanxi, China; State Key Laboratory of Crop Stress Biology for Arid Areas, Northwest A&F University, Yangling, 712100 Shaanxi, China; College of Plant Protection, Northwest A&F University, Yangling 712100, Shaanxi, China; State Key Laboratory of Crop Stress Biology for Arid Areas, Northwest A&F University, Yangling, 712100 Shaanxi, China; College of Plant Protection, Northwest A&F University, Yangling 712100, Shaanxi, China; State Key Laboratory of Crop Stress Biology for Arid Areas, Northwest A&F University, Yangling, 712100 Shaanxi, China; College of Plant Protection, Northwest A&F University, Yangling 712100, Shaanxi, China; State Key Laboratory of Crop Stress Biology for Arid Areas, Northwest A&F University, Yangling, 712100 Shaanxi, China; College of Plant Protection, Northwest A&F University, Yangling 712100, Shaanxi, China; State Key Laboratory of Crop Stress Biology for Arid Areas, Northwest A&F University, Yangling, 712100 Shaanxi, China; College of Life Sciences, Shanxi Normal University, Taiyuan 030000, Shanxi, China; College of Plant Protection, Northwest A&F University, Yangling 712100, Shaanxi, China; State Key Laboratory of Crop Stress Biology for Arid Areas, Northwest A&F University, Yangling, 712100 Shaanxi, China; College of Life Sciences, Shanxi Normal University, Taiyuan 030000, Shanxi, China; College of Plant Protection, Northwest A&F University, Yangling 712100, Shaanxi, China; State Key Laboratory of Crop Stress Biology for Arid Areas, Northwest A&F University, Yangling, 712100 Shaanxi, China; College of Plant Protection, Northwest A&F University, Yangling 712100, Shaanxi, China; State Key Laboratory of Crop Stress Biology for Arid Areas, Northwest A&F University, Yangling, 712100 Shaanxi, China; State Key Laboratory of Crop Stress Biology for Arid Areas, Northwest A&F University, Yangling, 712100 Shaanxi, China; College of Life Sciences, Northwest A&F University, Yangling 712100, Shaanxi, China

## Abstract

Remorins, plant-specific proteins, have a significant role in conferring on plants the ability to adapt to adverse environments. However, the precise function of remorins in resistance to biological stress remains largely unknown. Eighteen *CaREM* genes were identified in pepper genome sequences based on the C-terminal conserved domain that is specific to remorin proteins in this research. Phylogenetic relations, chromosomal localization, motif, gene structures, and promoter regions of these remorins were analyzed and a remorin gene, *CaREM1.4*, was cloned for further study. The transcription of *CaREM1.4* in pepper was induced by infection with *Ralstonia solanacearum*. Knocking down *CaREM1.4* in pepper using virus-induced gene silencing (VIGS) technologies reduced the resistance of pepper plants to *R. solanacearum* and downregulated the expression of immunity-associated genes. Conversely, transient overexpression of *CaREM1.4* in pepper and *Nicotiana benthamiana* plants triggered hypersensitive response-mediated cell death and upregulated expression of defense-related genes. In addition, CaRIN4-12, which interacted with CaREM1.4 at the plasma membrane and cell nucleus, was knocked down with VIGS, decreasing the susceptibility of *Capsicum annuum* to *R. solanacearum*. Furthermore, CaREM1.4 reduced ROS production by interacting with CaRIN4-12 upon co-injection in pepper. Taken together, our findings suggest that CaREM1.4 may function as a positive regulator of the hypersensitive response, and it interacts with CaRIN4-12, which negatively regulates plant immune responses of pepper to *R. solanacearum.* Our study provides new evidence for comprehending the molecular regulatory network of plant cell death.

## Introduction

Remorins (REMs) are plant-specific proteins that are typically marker proteins of the plasma membrane (PM) in all land plants, including ferns and mosses [1, 2]. The remorin protein was first detected in potato (*Solanum tuberosum*) in 1989, and was named pp34 because of its molecular weight of 34 kDa [3]. The protein was later renamed remorin to demonstrate its ability to associate with the PM [4]. Whole-genome sequencing projects analyzed some plant remorin genes. According to the forecast, there are 2, 4, 8, 11, 16, 19, and 20 remorin genes in *Welwitschia mirabilis*, *Physcomitrella patens*, poplars (*Populus* spp.), foxtail millet (*Setaria italica*), *Arabidopsis thaliana*, rice (*Oryza sativa*), and wheat (*Triticum aestivum*) genomes, respectively [1, 5, 6]. Remorin proteins contain a variable N-terminal and a conserved C-terminal domain, where the N-terminus is primarily responsible for structural and functional differences, while the C-terminus is critical for oligomerization and localization in the PM, including the coiled-coil motif [7]. Based on the different N-terminal domains and phylogenetic tree analysis, REMs were divided into six groups, of which groups 1, 2, and 3 were not phylogenetically distinguished but subdivided by domain characteristics [1].

REMs take part in plant growth, development, responses to abiotic and biotic stresses, signal transduction, and fruit ripening [1, 8–11]. Studies have revealed that REM proteins enhance plant resistance to bacteria, fungi, and viruses [12–15].StREM1.3 binds cell wall-derived galacturonides [16] and interacts with the viral protein TGBp1 [12], impairing the movement of virus X into potato [14]. *A. thaliana* remorin1.3 (AtREM1.3)is phosphorylated to varying degrees upon treatment with a bacterial elicitor [17, 18]. AtREM1.2 has been identified to be a RIN4 interacting protein, which is a negative regulator of plant immunity [19]. Furthermore, MtREM2.2 regulates bacterial infection by interacting with symbiotic receptors after phosphorylation by receptor-like kinase (RLK) [13]. Transgenic maize overexpressing the *ZmREM1.3* gene has been reported to show increased resistance to the *Puccinia polysora*. On the contrary, transgenic plants with *ZmREM1.3* gene mutation were more likely to be infected with *P*. *polysora* than control plants, suggesting that*ZmREM1.3* positively regulates the defense response of maize to *P*. *polysora* [20]. Fine mapping and expression and mutation analysishave shown that *ZmREM6.3* regulates quantitative resistance of maize to northern leaf blight [21]. MtSYMREM1 is a remorin family protein that interacts with symbionts to regulate pathogen infection during nodulation in *Medicago truncatula* [13]. SomeREM genes play negative regulatory roles in disease resistance.AtREM4.1 interacts with SnRK1 protein, resulting in AtREM4.1 phosphorylation and degradation by the 26S proteasome pathway, and plays a key role in imparting susceptibility to beet roll top virus in *A. thaliana* [22]. Studies have shown that overexpression of *SlREM1.3* in tomato plants enhances susceptibility to *Phytophthora infestans* [23]. In addition, REMs have been showed to play a significant role in responses to abiotic stresses [1, 9, 24], such as cold, salt, and drought stress [1, 6]. Moreover, *SlREM1* positively regulated fruit ripening in *SlREM1* overexpression and RNA interference (RNAi) lines [10]. Overexpression of *SlREM1.3* in tomato (*Solanum lycopersicum*) promoted leaf senescence [12].

Programmed cell death (PCD) is a basic biological process that leads to the natural suicide of cells [25, 26]. Interaction between plant and microorganism usually causes hypersensitive response (HR)-triggered PCD in plant host cells; this response is correlated with increased resistance to some pathogens [27]. PCD onset in plants is similar to biochemical and morphological traits of animal apoptosis, but there are some differences between the two forms of PCD [28]. Plant PCD is an intricate genetically programmed process. Reactive oxygen species (ROS) have been shown to perform a crucial function in regulating cell death [29], senescence [30], and resistance response in plants [31]. Increased ROS levels affect cellular components and trigger cell necrosis, while ROS at lower levels act as signaling molecules [29]. Cai *et al*. [32] report that transgenic tomatoes overexpressing *SlREM1 showed* enhanced susceptibility to *Botrytis cinerea*. In addition, transient expression of *SlREM1* in *Nicotiana benthamiana* induced PCD. ROS produced by respiratory burst oxidase homolog (RBOH) proteins play vital functions in cell death, particularly in the HR [33, 34]. Studies have reported that suppression of *N. benthamiana RBOH* genes reduced HR-like cell death induced by the protein stimulator INF1 [35]. Similarly, suppression of the *OsRBOHA* gene is known to significantly reduce HR in rice [36].

RPM1-interacting protein 4 (RIN4) plays an important role in both pathogen-associated molecular pattern (PAMP)-triggered immunity (PTI) and effector-triggered immunity (ETI) [37]. Previous studies have suggested that RIN4 functions as a negative regulator of PTI [38]. *In Arabidopsis*, *rin4* mutants show an enhanced defense response, which was contrary to observations in plants overexpressing *RIN4* [39]. *GmRIN4* genes have been reported to negatively regulate the basal resistance of soybean to *Pseudomonas syringae* and oomycete pathogens [40]. Moreover, RIN4 can be targeted and modified by several different bacterial effectors and can interact with plant RPM1 (resistance to *P. syringae* pv. *maculicola* 1), RPS2 (resistance to *P. syringae* 2), and some other R proteins [38, 41, 42]. Recent studies have shown that RIN4 may control exocytosis to confer plant immunity. EXO70 protein promotes callose deposition in the cell wall in the presence of flg22. RIN4 can promote the transport of EXO70E2 vesicles [43], indicating that RIN4 plays a positive role in the basic defense of plants. Additionally, the results of mass spectrometry suggested that REM protein belongs to the RIN4 protein complex in *A. thaliana* [19]. In *Populus euphratica*, PeREM6.5 interacted with PeRIN4 to regulate the activity of PM H^+^-ATPase [24]. However, despite these advances, REMs and RIN4 members still need to be further studied with regard to regulating the immune response of pepper (*Capsicum annuum* L.) and *Ralstonia solanacearum*.

Pepper, a Solanaceae plant with huge agricultural importance, has important economic and edible value, but it is susceptible to infection by *R. solanacearum*, which can have serious impacts on the quality and yield of pepper. *R. solanacearum* could cause bacterial wilt disease, which is a serious pathogenic bacterium in Solanaceae crops, including pepper, tomato, and potato [44, 45]. Despite the commercial importance of *C. annuum*, the defense mechanisms of peppers against pathogens are largely unknown. To date, the functional and expression characteristics of very few REM genes in Solanaceae crops are known. However, the exact function of most REMs in pepper remains to be further investigated.

In this study, 18 *CaREM* genes of pepper were identified based on the C-terminal conserved domain of REM proteins. Phylogenetic analysis, chromosomal localization, motif, gene structure, and promoter of these REMs were investigated. In addition, *CaREM1.4*, a member of the REM family of peppers, was used as the target to preliminarily analyze the function of *CaREM* in plant responses to pathogens by analyzing the interaction system of pepper and the *R. solanacearum* pathogen. The transcription of *CaREM1.4* in pepper was induced by *R. solanacearum*. Knocking down *CaREM1.4* expression by virus-induced gene silencing (VIGS) attenuated *R. solanacearum* infection intensity and reduced the accumulation of ROS, triggering cell death. Moreover, CaRIN4-12, which was an immune negative regulator that interacted with CaREM1.4, was knocked down with VIGS. This enhanced the disease resistance of silenced pepper compared with the control. These results suggested that CaREM1.4 is a positive regulator of HR that interacts with CaRIN4-12, which negatively regulates plant immune responses of pepper to *R. solanacearum*.

## Results

### Identification and phylogenetic analysis of the pepper remorin gene family

**Figure 1:**
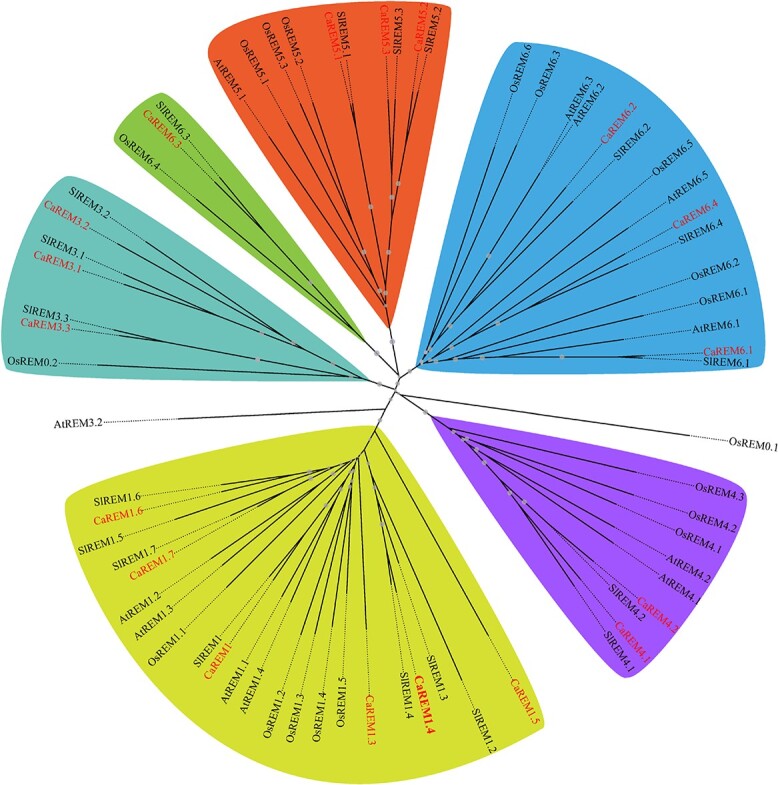
. Neighbor-joining phylogenetic analysis of remorin proteins in *C. annuum*, *S. lycopersicum*, *O. sativa*, and *A. thaliana*. Bootstrap values (1000 replicates) are shown for each branch. Pepper proteins are indicated in red. Different colors are used to highlight different groups, and the points on the branches represent the bootstrap support values.

To identify REM genes from *C. annuum*, the Ensembl database was searched using the conserved sequence at the C- terminal of REM as a query. Based on the search results, 18 complete *CaREM* coding sequences were identified. In total, 18 *CaREM* genes were identified according to their characteristic information, including the gene ID, chromosome location number of the ORF (open reading frame), amino acid length, introns, exons, isoelectric point, and molecular weight and so on (Table 1). The results showed that predicted protein sizes of the encoded CaREMs ranged from 119 to 571 amino acids and isoelectric points were 5.29–10.03. Multi-sequence alignment of the C-terminal domains of 18 pepper REM proteins showed that they all contain a conserved coiled-coil domain (Supplementary Data Fig. S1). To study the phylogenetic relationships among the proteins belonging to the REM family in pepper, we retrieved the 16 *A. thaliana* REM members [1], 18 tomato REM proteins, and 19 rice REM members to construct evolutionary trees (Supplementary Data Table S1). The 18 *CaREM* genes identified above were divided into five groups (groups 1, 3, 4, 5, and 6) (Fig. 1). We assigned identical gene numbers to orthologs of pepper according to the method of Raffaele *et al*. [1].

**Table 1:** Characteristics of REM genes in pepper identified from the genome-wide search analysis.

Gene name	Gene ID	Chromosome location	ORF (bp)	Total amino acid length	Intron no.	Exon no.	pI	Mol wt (Da)	Subcellular localization
*CaREM 1.1*	PHT85954	25 421 566–25 423 804	633	210	4	5	5.29	23201.63	Nucleus; cytoplasm
*CaREM 1.3*	PHT92456	5 704 749–5 708 489	591	196	4	5	5.97	22253.30	Cytoplasm
*CaREM 1.4*	PHT78353	87 116 738–87 119 705	549	180	4	5	6.68	19811.88	Nucleus; cytoplasm
*CaREM 1.5*	PHT70022	46 144 559–46 149 787	504	167	4	5	9.77	18205.06	Nucleus
*CaREM 1.6*	PHT74495	1 246 930 283–124 696 039	510	169	4	5	9.33	18562.32	Nucleus; cytoplasm
*CaREM 1.7*	PHT81820	184 934 601–184 937 247	360	119	2	3	9.75	13661.80	Nucleus
*CaREM 3.1*	PHT67705	18 363 661–18 365 709	1143	380	5	6	9.69	42970.85	Nucleus
*CaREM 3.2*	PHT92918	17 582 638–17 584 689	837	278	5	6	10.03	31108.35	Nucleus; cytoplasm
*CaREM 3.3*	PHT79616	224 378 399–224 379 269	591	196	3	4	10.01	22337.62	Nucleus; cytoplasm
*CaREM 4.1*	PHT80637	1 563 312–15 667 858	888	295	1	2	6.22	33360.60	Nucleus
*CaREM 4.2*	PHT71383	230 085 276–230 088 869	882	293	1	2	9.10	33165.38	Nucleus
*CaREM 5.1*	PHT63212	300 480–305 190	1716	571	6	7	9.63	62591.13	Nucleus
*CaREM 5.2*	PHT89469	91 387 409–91 390 689	1161	386	3	4	9.53	42906.95	Cytoplasm
*CaREM 5.3*	PHT90001	130 030 063–130 032 307	1155	360	3	4	9.77	39290.68	Nucleus
*CaREM 6.1*	PHT73899	1 195 548–1 200 397	1572	523	5	6	8.78	58254.58	Nucleus
*CaREM 6.2*	PHT74124	47 340 784–47 345 928	1575	524	5	6	9.03	58381.06	Cytoplasm
*CaREM 6.3*	PHT88896	281 704 282–281 707 074	1431	476	4	5	7.94	53503.17	Cytoplasm
*CaREM 6.4*	PHT66708	241 851 475–241 852 783	1083	360	2	3	9.39	40065.02	Nucleus

pI, isoelectric point; Mol wt, molecular weight.

### Chromosomal position and structure analysis of putative *CaREM* genes

The 18 *CaREM* genes of pepper identified here were distributed across 10 of the 12 chromosomes, with different distribution densities on different chromosomes (Supplementary Data Fig. S2). For example, chromosomes 2 and 8 contained three *CaREM* genes, chromosomes 1, 3, 5, 6, and 10 had two *CaREM* genes, while chromosomes 11 and 12 had one *CaREM* gene. The specific position of each REM gene on the chromosome is presented in Table 1.

The exon and intron structures of the *CaREMs* were analyzed to further understand the evolution of *CaREM*s (Table 1). Our analysis showed intron number to be conserved and ranged from one to six per gene in pepper. The genes from phylogenetic groups 1 (*CaREM1*, *CaREM1.3*, *CaREM1.4*, and *CaREM1.5*) and 4 (*CaREM4.1* and *CaREM4.1*) had four introns and one intron, respectively. *CaREM6.1* and *CaREM6.2* contained five introns, while *CaREM3.1* and *CaREM3.2* had five introns. The conserved number of introns suggests that REM genes in pepper have similar gene structure diversity. Most gene sequences in the same group generally have similar homology, and the same number of introns, indicating that *CaREM* genes are evolutionarily conserved, and there is related gene structure diversity.

### Motif and promoter analysis of the putative *CaREM* genes

To estimate the diversity and conservation of the CaREMs, motifs were recognized through the MEME program. Ten distinct motifs among the 18 CaREM proteins were identified. The distribution of CaREM protein motifs in the different groups and the identification of the consensus sequence of *CaREM*s are shown in Supplementary Data Fig. S3 and Supplementary Data Table S2. The identified motifs ranged in size from 24 to 41 amino acids (Supplementary Data Table S2). Generally speaking, REM proteins have a variable N-terminal region and a C-terminal domain that is conservative. Motif analysis showed that all CaREM proteins had the same C-domain (motif 1), indicating their evolutionary conservation in plants. The transcriptional response elements of 18 *CaREM* promoters were analyzed using the PlantCARE promoter database to further elucidate their regulatory mechanisms. We found that the promoters of 18 *CaAREMs* had 15 *cis*-acting elements, including the W-box, ERE, CAAT-box, TCA-element, TC-rich repeats, TATA-box, TGACG-motif, P-box, MBS, CGTCA-motif, ABRE, CCGTCC-box, A-box, TATC-box, and LTR (Supplementary Data Table S3).

### *CaREM1.4* transcript increases in pepper leaves exposed to *R. solanacearum*

**Figure 2:**
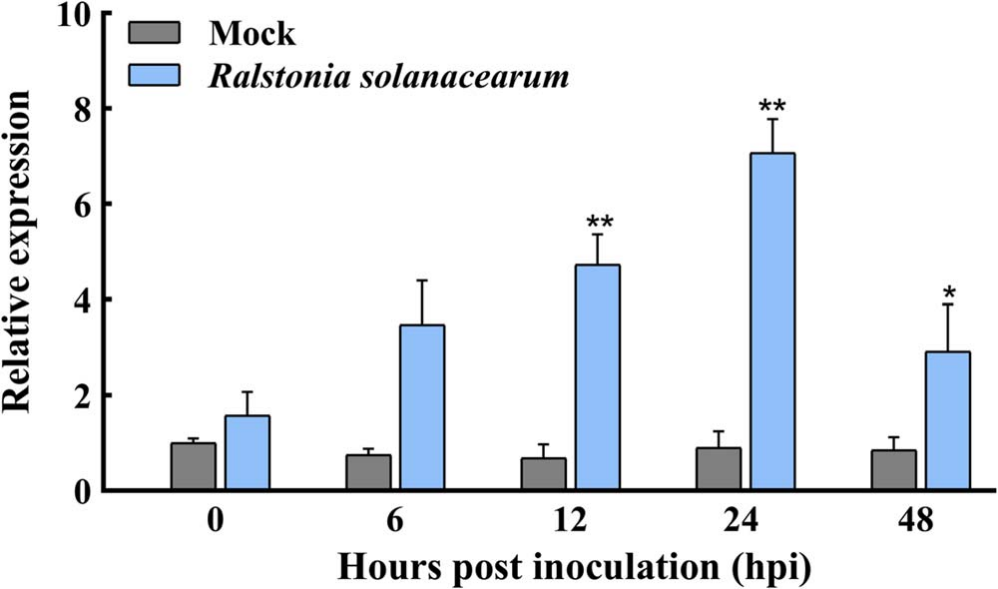
qRT–PCR analysis of relative transcript levels of pepper *CaREM1.4* under treatment for *R. solanacearum*. The third leaves of pepper plants were infected with *R. solanacearum* strain Px1 (phylotype I) (OD_600_ = 0.8) and the mock treatment was injection with 10 mM MgCl_2_. The expression level of *CaREM1.4* in untreated plants was set to 1. *CaActin* was used as an internal reference gene. Data represent the means ± standard deviations from three independent values.^ **^P < .01, significant difference compared with control by Student’s *t* test.

It has been reported that multiple SlREM proteins in subgroup I are involved in the plant immune response [32]. Quantitative real-time PCR (qRT–PCR) was used to analyze the transcriptional changes of *CaREM*s belonging to subgroup I in response to *R. solanacearum* stress (Supplementary Data Fig. S4). The upregulated express ion of *CaREM1.4* after *R. solanacearum* treatment for further analysis because this expression was more significant. The promoter of *CaREM1.4* contains *cis*-acting elements related to the regulation of the immune response, including the TGACG motif [46, 47], ERE-box [48], ABRE-box [48], and W-box [49], suggesting that the gene may be related to the response to pathogen infection. To verify the hypothesis, the transcriptional level of *CaREM1.4* in leaves of pepper plants after inoculation with *R. solanacearum* was determined by qRT–PCR (Fig. 2). Specific primer sequences of qRT–PCR are shown in Supplementary Data Table S4. Compared with the mock treatment, the expression level of *CaREM1.4* was significantly increased at 12 and 24 hours post-inoculation (hpi) after pepper leaves were injected with *R. solanacearum* strain, Px1 (phylotype I). The results revealed that *CaREM1.4* was upregulated in response to the induction of *R. solanacearum*.

### Silencing of C*aREM1.4* impairs resistance of pepper to *R. solanacearum*

**Figure 3:**
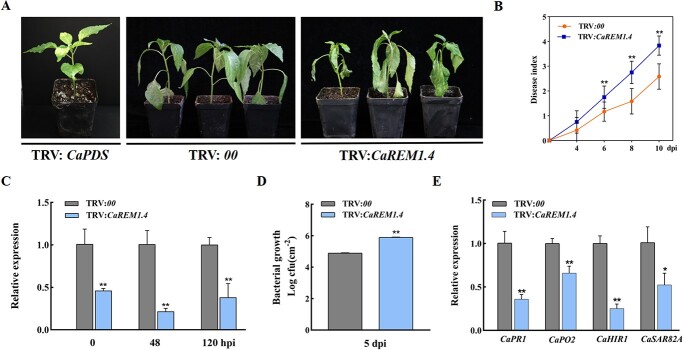
*CaREM1.4*-silenced pepper plants reduced resistance to *R. solanacearum*. (A) Phenotypes of *CaREM1.4*-knockdown and control plants at 8 dpi after *R. solanacearum* infection. (B) Disease index score was recorded from 4 to 10 days after wounded root inoculation with *R. solanacearum*. Each point represents the mean disease index of 12 inoculated plants. (C) Relative transcriptional level of *CaREM1.4* in TRV:*CaREM1.4* pepper plants compared with TRV:*00* plants by qRT–PCR at 0, 48, and 120 hours after leaf inoculation with *R. solanacearum*. (D) *R. solanacearum* growth in control or *CaREM1.4*-silenced plants at 5 dpi. (E) Relative expression of immunity-related genes in TRV:*00* and TRV:*CaREM1.4* plants. In B, C, D and E, values represent the means ± standard deviations of three independent samples. ^*^P < .05, ^**^P < .01, significant difference compared with control by Student’s *t* test.

The transcriptional level of *CaREM1.4* was upregulated by *R. solanacearum*, indicating that *CaREM1.4* may be important in pepper immunity. Therefore, we used the tobacco rattle virus (TRV) VIGS system to further research the role of *CaREM1.4* in the pepper–*R. solanacearum* interaction (Fig. 3). A 315-bp specific fragment of *CaREM1.4* in the coding sequence was selected for construction of the VIGS vector, pTRV2-CaREM1.4 (Supplementary Data Fig. S5). A total of 30 plants without silencing of *CaREM1.4* (TRV:*00*) and 30 plants with silencing of *CaREM1.4* (TRV:*CaREM1.4*) were obtained. When the bleaching phenotype was observed in phytoene desaturase (PDS)-silenced plants, indicating that *CaREM1.4* was effectively silenced, peppers were inoculated with the *R. solanacearum*-compatible strain Px1 (phylotype I) by root irrigation. *CaREM1.4-*unsilenced plants showed a very weak wilting phenotype, while *CaREM1.4-*silenced plants (Fig. 3A) showed significant wilting disease symptoms at 8 days post-inoculation (dpi). The relative disease index was determined from 4 to 10 dpi for *CaREM1.4-*silenced and control plants (Fig. 3B). The silencing efficiency of *CaREM1.4* was determined using qRT–PCR at 0, 48, and 120 hpi of *R. solanacearum* infection. The transcription level of *CaREM1.4* was reduced by 17–60% compared with the control (Fig. 3C). Meanwhile, the transcript levels of other subgroup I *CaREM*s in *CaREM1.4*-knockdown plants were confirmed by qRT–PCR, showing no significant change compared with the control (Supplementary Data Fig. S6). These results for silencing efficiency indicated that only *CaREM1.4* was significantly knocked down. In addition, the increased susceptibility of pepper to the pathogens was manifested in increased *R. solanacearum* population growth. Compared with the non-silenced plants, the number of colony forming units (CFU) of pepper plants with *CaREM1.4* silenced was higher at 5 dpi (Fig. 3D).

Transcription levels of pepper defense-associated genes, including *CaPR1*, *CaPO2*, *CaHIR1*, and *CaSAR82A*, were detected in TRV:*CaREM1.4* and TRV:*00* pepper plants (Fig. 3E). Upon challenge by *R. solanacearum*, the transcriptional levels of *CaPR1*, *CaPO2*, and *CaHIR1* in TRV:*CaREM1.4* plants were significantly reduced compared with those in control. These results show that *CaREM1.4* performed a positive regulatory function in the pepper–*R. solanacearum* interaction.

### Transient overexpression of *CaREM1.4* triggers cell death and upregulates immunity-associated genes in *C. annuum* and *N. benthamiana*

**Figure 4:**
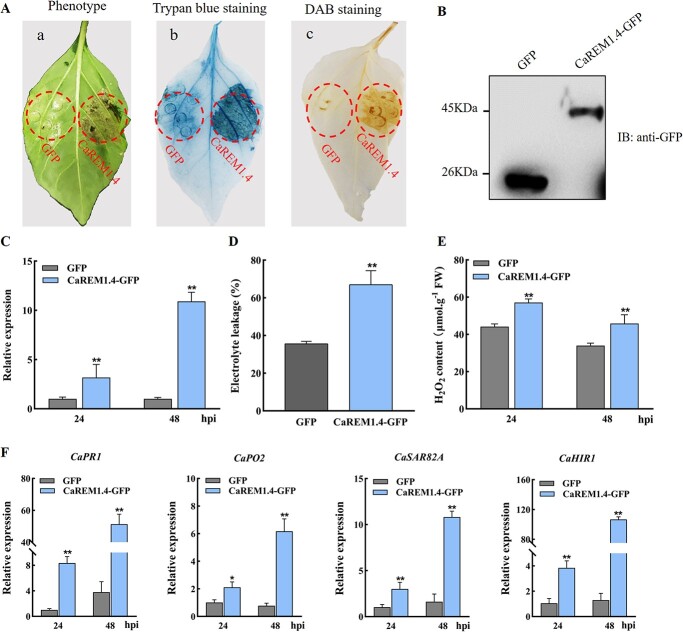
Transient expression of *CaREM1.4* in pepper leaves triggered cell death and ROS accumulation. (A) Phenotypic observation of pepper leaves infiltrated with *A. tumefaciens* GV3101 carrying GFP and CaREM1.4-GFP vector. (a–c) Phenotype of leaves at 4 dpi (a), after staining with trypan blue to assess cell death (b), and after staining with DAB to detect hydrogen peroxide (c). Red circles indicate infiltration areas. (B) Immunoblotting test of CaREM1.4 protein in pepper leaves; the antibody was anti-GFP. IB, immunoblotting. (C) Transient *CaREM1.4* overexpression was determined by qRT–PCR. (D) Quantification of electrolyte leakage to evaluate cell death in leaf disks at 48 hpi. (E) Accumulation of H_2_O_2_ in pepper leaves overexpressing GFP and CaREM1.4-GFP at 24 and 48 hpi; (F) Expression of defense-associated marker genes in *CaREM1.4*-expressing pepper leaves at 24 and 48 hpi. In C, D, E, and F, values represent the means ± standard deviations of three independent samples. ^**^P < .01, significant difference compared with control by Student’s *t* test.

To further clarify that *CaREM1.4* plays a positive regulatory role in the pepper–*R. solanacearum* interaction, the *CaREM1.4* gene was instantaneously expressed in pepper plants by injecting *Agrobacterium tumefaciens* containing CaREM1.4-GFP plasmids. Transient *CaREM1.4* overexpression stimulated cell death after 4 dpi. However, there were no apparent necrotic phenotypes in control plants (Fig. 4A, a). HR-associated cell death was observed by trypan blue staining, indicating that brief overexpression of *CaREM1.4* induced a significant necrosis response in pepper leaves, while only a mild HR-mediated necrosis response was observed in leaves injected with *A. tumefaciens* GV3101 cells carrying empty vectors (Fig. 4A, b). Moreover, leaves with transient expression of CaREM1.4-GFP were stained with DAB (3,3-diaminobenzidine) to detect H_2_O_2_ production, while control leaves were not stained (Fig. 4A, c). *CaREM1.4* was successfully expressed in western blotting assay (Fig. 4B) and qRT–PCR (Fig. 4C) experiments. The results showed that *CaREM1.4* was successfully expressed. In the meantime, we also measured the ion leakage of leaves expressing CaREM1.4-GFP to analyze the effect of PM injury on cell necrosis, and found that pepper leaves with CaREM1.4-GFP agroinfiltration had more ion leakage at 48 hpi than those with GFP (Fig. 4D). Furthermore, the cytosolic levels of ROS were investigated in pepper leaves. Coincidentally, the H_2_O_2_ content of leaves expressing CaREM1.4-GFP increased significantly compared with pepper leaves expressing GFP (Fig. 4E). The transcriptional expression of immune marker genes, including *CaPR1*, *CaHIR1*, *CaPO2*, and *CaSAR82A*, was examined. These results showed that overexpression of *CaREM1.4* could increase the relative expression of these defense-associated genes in plants (Fig. 4F).

CaREM1.4 protein consists of 183 amino acids, including an N-terminal and a conserved C-terminal region (Supplementary Data Fig. S7A). To further identify which domains in CaREM1.4 are necessary for cell death, a set of GFP-tagged CaREM1.4 truncates were constructed: the ORF of 183 amino acids (CaREM1.4 1–183), the N-terminal region (CaREM1.4-N, 12–67), and the C-terminal region (CaREM1.4-C, 70–176), were infiltrated into pepper leaves by agroinfiltration. The phenotype of tobacco leaves was examined at 6 dpi, and the results showed that the C-terminal domain was necessary for cell death (Supplementary Data Fig. S7B). Both the N-terminus and the C-terminus of protein CaREM1.4 could be localized on the cell membrane, and the C-terminus of CaREM1.4 was also noted to be located in the nucleus (Supplementary Data Fig. S7C).

Furthermore, *CaREM1.4* was transiently overexpressed in tobacco leaves. Compared with the control, there was a significant necrotic phenotype in tobacco leaves overexpressing the *CaREM1.4* gene (Supplementary Data Fig. S8A). The expression of *CaREM1.4* was demonstrated by qRT–PCR and western blotting with GFP antibody (Supplementary Data Fig. S8B and C). In addition, the transcript levels of defense-related genes were detected in CaREM1.4-expressing *N. benthamiana* leaves at 24 and 48 hpi. The expression of *NbPR1*, *NbHSR201*, and *NbHSR2505* was enhanced, but the expression of *NbPOD* was reduced in CaREM1.4-GFP. (Supplementary Data Fig. S8D). These results are identical to the effects of *CaREM1.4* observed in pepper plants.

### Interaction of CaREM1.4 and CaRIN4-12

RIN4 negatively regulates plant PTI and ETI responses [50, 51]. Previous studies showed that PeREM6.5 interacted with PeRIN4 to regulate the activity of PM H^+^-ATPase in *Populus euphratica*, suggesting that CaREM1.4 may interact with RIN4 in pepper. To acquire *RIN4* genes from *C. annuum*, the NCBI database was used for searching as a reference genome. Based on the search results, 13 complete CaRIN4 coding sequences were identified. Thirteen *C. annuum* RIN4 proteins and 25 *A. thaliana* RIN4 proteins were used to construct the phylogenetic tree (Supplementary Data Table S1). The 13 CaRIN4s were divided into four groups (groups I, II, IV, and V) (Supplementary Data Fig. S9). Split-luciferase (Split-LUC) assays showed that CaREM1.4 can interact with four members of the CaRIN4 family (Supplementary Data Fig. S10). These members represent different groups of the RIN4 family and contain conserved sequences, and we speculate that CaREM1.4 can interact with RIN4 proteins. To further verify the reliability of interaction, *CaRIN4-12*, a homologue of *PeRIN4*, was cloned in *C. annuum* ‘Zhongjiao’. The *CaRIN4-12* gene was selected for subsequent interaction and functional verification.

**Figure 5:**
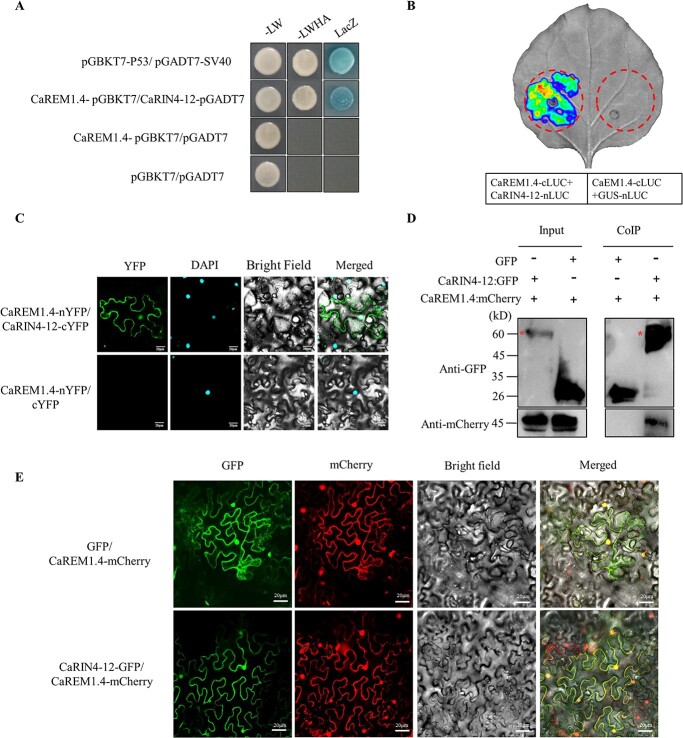
CaREM1.4 interacted with CaRIN4-12. (A) Interaction between CaREM1.4 and CaRIN4-12 was examined in a Y2H assay. -LW, yeast growth on medium without Leu and Trp; -LWHA, yeast growth on medium lacking Leu, Trp, His, and Ade; LacZ, activity of the lacZ reporter gene. (B) Split-LUC assays determined the interaction of CaREM1.4 and CaRIN4-12 in *N*. *benthamiana* leaves. GUS-nLUC was used as the negative control. (C) Interaction of CaREM1.4 and CaRIN4-12 was verified by BIFC analysis. nYFP-CaREM1.4/CaRIN4-12-cYFP was the experimental group, and nYFP-CaREM1.4/cYFP was the negative control. YFP fluorescence was observed by confocal microscopy. DAPI was used as a nuclear dye. Scale bar = 20 μm. (D) Protein interactions of CaREM1.4-mCherry and CaRIN4-12-GFP were examined by Co-IP analysis. Anti-mCherry and anti-GFP were used to detect protein expression (GFP as a control). Red asterisks indicate the location of the target protein. (E) Co-localization between CaREM1.4 with CaRIN4-12. CaREM1.4-mCherry was co-expressed with GFP or CaRIN4-12-GFP in *N. benthamiana* leaves at 48 hpi. The *Agrobacterium* concentration OD_600_ = 0.6. Scale bar = 20 μm.

The interaction between CaREM1.4 and CaRIN4-12 was tested by yeast two-hybrid (Y2H), Split-LUC complementation, bimolecular fluorescence complementation (BIFC), and co-immunoprecipitation (Co-IP) assays. We first verified the interaction between CaREM1.4 and CaRIN4-12 by yeast Y2H assays. AH109 strains co-expressing CaREM1.4-pGBKT7 and CaRIN4-12-pGADT7 could grow on yeast defective medium and show β-galactosidase activity (Fig. 5A). The interaction between them was further confirmed by Split-LUC assays. Only the region co-injected with CaREM1.4-cLUC and CaRIN4-12-nLUC had strong luminescent signals. No luminescent signals were found in the region co-expressing CaREM1.4-cLUC and GUS-nLUC (Fig. 5B). CaREM1.4 and CaRIN4-12 coding sequences were inserted into pSPYNE(R)173 and pSPYCE(M) vectors, respectively, and infiltrated into tobacco leaves. The physical association between them was further detected by the BIFC method. The fluorescent signals of the interaction between CaREM1.4 and CaRIN4-12 were detected under the microscope after 48 hpi; they were specifically observed in the PM and nucleus. By contrast, no fluorescent signals were observed in the control when co-expressed with CaREM1.4-nYFP and cYFP (Fig. 5C). To validate the interaction between CaREM1.4 and CaRIN4-12 in plants, we performed Co-IP assays. All proteins were tested in the input group, and CaREM1.4-mCherry was co-immunoprecipitated only when CaRIN4-12 was expressed, but not in the negative control GFP (Fig. 5D). In conclusion, these results suggested that CaREM1.4 interacted with CaRIN4-12 in the cell membrane and nucleus.

REMs are PM-associated proteins found in all embryophytes [9]. Although REM proteins are demonstratively related to the PM, they lack transmembrane domains. The PSORT program predicted that CaREM1.4 was localized in the nucleus and cytoplasm. To clarify the functional location of the interaction between CaREM1.4 and CaRIN4-12, we co-expressed GFP/CaRIN4-12-GFP and CaREM1.4-mCherry in *N. benthamiana* leaves (Fig. 5E). The results indicated that CaREM1.4 and CaRIN4-12 were co-located on the cell membrane and nucleus.

To further analyze the key domain of CaREM1.4 interaction with CaRIN4-12, Split-LUC and BIFC assays were used to verify the interaction between the C- or N-terminal domain of CaREM1.4 and CaRIN4-12 in *N. benthamiana* leaves. The results showed that CaRIN4-12 interacts with only the C-terminal domain of CaREM1.4, but not the N-terminal domain (Supplementary Data Fig. S11).

### *CaRIN4-12* reduced reactive oxygen species and cell death produced by *CaREM1.4*

**Figure 6:**
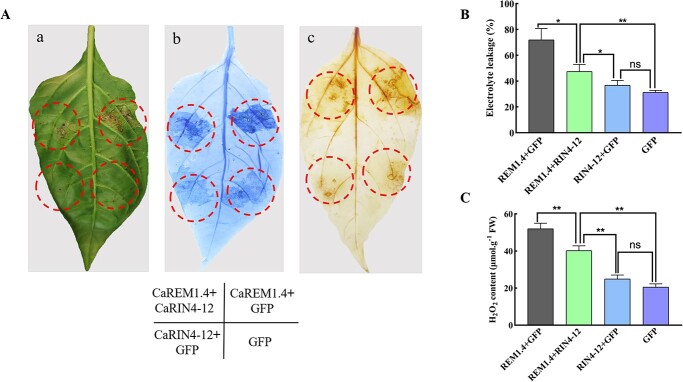
Co-expression of *CaREM1.4* and *CaRIN4-12* in pepper leaves reduces ROS and necrosis. (A) Pepper leaves were infiltrated with *A. tumefaciens* carrying CaREM1.4-GFP + GFP, CaREM1.4-GFP + CaRIN4-12-GFP, CaRIN4-12-GFP + GFP and GFP (empty vector). (a–c) Phenotypes of infiltrated pepper leaves at 4 days after agroinfiltration (a), by trypan blue staining (b), and by DAB staining (c). (B) Quantification of electrolyte leakage (ion conductivity) to evaluate the cell death response in leaf disks at 48 hpi. (C) Accumulation of H_2_O_2_ in pepper leaves transiently expressing GFP-CaREM1.4/GFP, GFP-CaREM1.4/GFP-CaRIN4-12, GFP-CaRIN4-12/GFP, and GFP at 48 hpi. In B and C values represent the means ± standard deviations of three independent samples. ^*^P < .05, ^**^P < .01, significant difference compared with control by Student’s *t* test.

Previous experiments have shown that overexpression of *CaREM1.4* can cause ROS accumulation and cell death. To further study the mechanism of the interaction between CaREM1.4 and CaRIN4-12 in regulating the plant immune response, we co-injected CaREM1.4-GFP and CaRIN4-12-GFP into pepper leaves. CaREM1.4-GFP + GFP was a positive control and GFP empty vector was a negative control. The results showed that, compared with the positive control, the necrosis phenotype after co-injection was significantly weaker than that after injection alone (Fig. 6A). There was significantly less ion leakage in leaves that were transiently overexpressing CaREM1.4-GFP + CaRIN4-12-GFP compared with CaREM1.4-GFP + GFP leaves 48 hours after agro-infiltration of GV3101 strains (Fig. 6B). Transient overexpression of CaRIN4-12-GFP + GFP leaves showed no significant difference in ion leakage compared with GFP alone. In addition, ROS content was quantified by measuring the accumulation of H_2_O_2_ in pepper leaves. The H_2_O_2_ content in leaves that co-expressed CaREM1.4-GFP + CaRIN4-12-GFP was obviously lower than that of the control. It was higher than in leaves that were injected with the CaRIN4-12-GFP + GFP or GFP empty vector (Fig. 6C). These results suggest that *CaRIN4-12* reduced the ROS and cell death produced by *CaREM1.4* significantly.

### *CaRIN4-12* silencing enhances the resistance of pepper to *R. solanacearum*

**Figure 7:**
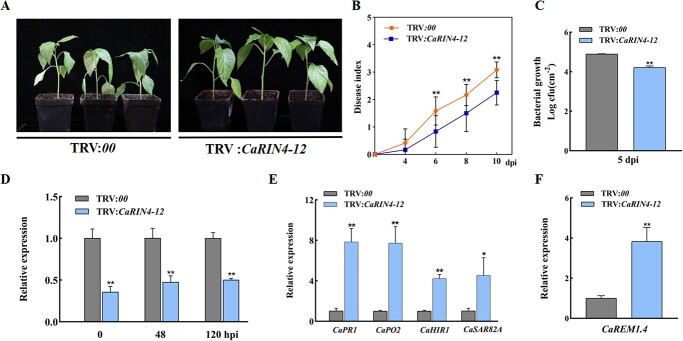
VIGS of *CaRIN4-12* decreased susceptibility of pepper to *R. solanacearum.* (A) Phenotypes of *CaRIN4-12*-knockdown and non-knockdown pepper plants at 8 dpi after *R. solanacearum* infection. (B) Dynamic disease index of TRV:*00* and TRV:*CaRIN4-12* pepper plants from 4 to 10 dpi. The averages presented are based on three biological replicates each comprising 12 plants. (C) *R. solanacearum* growth in control or *CaRIN4-12-*silenced pepper plants at 5 dpi. (D) Relative transcriptional level of *CaRIN4-12* in control and *CaRIN4-12-*silenced plants at 0, 48, and 120 hpi by qRT–PCR. (E) Relative expression of immunity-related genes in TRV:*00* and TRV:*CaRIN4-12* plants. (F) Relative transcription level of *CaREM1.4* in *CaRIN4-12*-knockdown pepper plants compared with TRV:*00* plants. In B, C, D, E, and F, values represent the means ± standard deviations of three independent samples. ^*^P < .05, ^**^P < .01, significant difference compared with control by Student’s *t* test.

To determine whether *R. solanacearum* infection can induce the expression of *CaRIN4-12*, transcriptional levels of *CaRIN4-12* after inoculation with *R. solanacearum* were determined by qRT–PCR (Supplementary Data Fig. S12). The results revealed that *CaRIN4-12* was upregulated in response to the induction of *R. solanacearum*. In order to further study the function of *CaRIN4-12* in the defense response of pepper against *R. solanacearum*, we conducted a functional loss test on pepper seedlings by performing VIGS on *CaRIN4-12*. A schematic diagram of *CaRIN4-12*’s VIGS vector construction is shown in Supplementary Data Fig. S5. Compared with the control plants, disease resistance in pepper leaves was enhanced after silencing *CaRIN4-12* at 8 dpi (Fig. 7A). Disease index of TRV:*CaRIN4-12* and TRV:*00* pepper plants was recorded from 4 to 10 days after infection with *R. solanacearum* (Fig. 7B). The bacterial growth in pepper leaves after *CaRIN4-12* gene silencing was remarkably lower than that in the control plants at 5 dpi (Fig. 7C). The qRT–PCR results showed that the relative expression of *CaRIN4-12* was significantly reduced in the TRV:*CaRIN4-12* plants compared with that in the control plants 0, 48, and 120 hours after infection with *R. solanacearum* (Fig. 7D). Moreover, we assayed the expression of immune-related genes in *CaRIN4-12*-knockdown plants (Fig. 7E). Transcript abundances of *CaPR1*, *CaPO2*, *CaHIR1*, and *CaSAR82A* were markedly higher in the *CaRIN4-12*-silenced plants than in the control. In addition, after silencing the *CaRIN4-12* gene the transcript levels of *CaREM1.4* were significantly upregulated (Fig. 7F). However, after the silencing of *CaREM1.4* the transcript abundances of the *CaRIN4-12* gene were unchanged (Supplementary Data Fig. S13). These results shown that knockdown of *CaRIN4-12* may increase the disease resistance of pepper against *R. solanacearum* and that *CaRIN4-12* negatively regulates the expression of *CaREM1.4*.

## Discussion

REMs are specific plant proteins that play significant roles in responses to biotic and abiotic stress factors. However, only a few *REM*s have been found in plants. Recently available genome sequences allow us to systematically study this gene family in *A. thaliana*, rice, potato, maize (*Zea mays*), and wheat. Nevertheless, only a few members of the REM family have been identified in vegetables, and they are especially associated with regulating the response of plants to biotic stress. Therefore, the biological function of *REM* genes in most vegetables need further study.

In the past, the 16 *A. thaliana REM* members were generally subdivided into six separate groups owing to the remarkable differences in their N-terminal domains [1]. To further explore the phylogenetic relationships of the *C. annuum REM* genes, we searched the 16 *A. thaliana* REM proteins [1], along with 19 and 18 *REM* members from *O. sativa* and *S. lycopersicum*. The 18 *CaREM* genes were also divided into five subfamilies (1, 3, 4, 5, and 6), which were the same as in *A. thaliana*. Phylogenetic analysis showed that 11 REMs from foxtail millet could be divided into four subgroups [5]. In wheat, 20 *TaREM*s were identified and divided into six phylogenetic groups [6]. The phylogenetic similarity between *C. annuum* and *A. thaliana* REM proteins suggested that they might have been conserved through evolution. In addition, the evolution of gene families can be further understood by analyzing the exon–intron structure and sequence of conserved regions. Studies found that the intron number of identified *REM* genes was highly variable. The diverse gene subfamilies and structures of the pepper *REM*s may reflect the different functions of these genes in biological and non-biological stress.

The subcellular localization of proteins is crucial for their function. REM proteins are widely recognized as marker proteins in the PM in all terrestrial plants and played a significant role in plant–microbe interactions [12, 32], whereas bioinformatics software predicted that 18 CaREM proteins were mainly localized in the cytoplasm and nucleus. In addition, some wheat REM proteins were located in the cytoplasm [6]. Previous studies demonstrated that the C-terminal region performs a decisive function in the lobule mechanism of the REM protein-specific binding membrane domain [52]. The N-terminal region varies greatly under various biological conditions [53]. In our study, CaREM1.4 was mainly localized to the cell membranes and nuclei. Furthermore, both the N- and C-terminal domains of CaREM1.4 could be localized in the PM, but only the C-terminal domains were necessary for nuclear localization, which showed that CaREM1.4 has various structures and functions. These differences may be connected with motif sequences, suggesting that the localization of CaREMs is complicated and varied. Moreover, some studies have shown that the C-terminal of StREM1.3 is essential for its PM localization [8, 54]. In a range of deletion mutants, only SlREM1 of 35 C-terminal amino acids was able to localize to the PM [32]. The specific amino acids in CaREM1.4 required for cell membrane localization remain to be further studied.

Plant cell death is critical for plant growth, evolution, and adaptability to the environment [29, 32]. The HR is a form of PCD that responds to pathogen attacks and is involved in resistance to biotrophic and semi-biotrophic pathogens [27, 55–57]. ROS play crucial functions in the regulation of PCD [27, 58], and can act as positive or negative regulators of PCD, depending on requirements and conditions [58–60]. *SlREM1* positively regulates PCD, which activates the burst of ROS in *N. benthamiana* leaves. The amino acids from 173 to 187 of SlREM1 have a crucial function in inducing cell death [32]. In this study, CaREM1.4 was involved in mediating plant cell death, where the C-terminal region of CaREM1.4 was necessary to trigger cell death because of its nuclear localization. The results of BIFC experiments showed that the C-terminal domain of CaREM1.4 and CaRIN4-12 interacted on the cell membrane instead of on the nucleus, indicating that CaRIN4-12 changed the nuclear localization of CaREM1.4 and reduced cell death by interacting with the C-terminal domain of CaREM1.4. As for the specific role of CaREM1.4, C-terminal amino acids in the middle stage of cell death are yet to be further studied. In addition, pepper plants with *CaREM1.4* knockout were highly susceptible to *R. solanacearum* infection; they were also impaired in ROS accumulation and hypersensitive cell necrosis in leaves. In contrast, overexpression of *CaREM1.4* in *C. annuum* and *N. benthamiana* triggered cell death, H_2_O_2_ accumulation, and induction of the expression of immune resistance genes. These results suggest that *CaREM1.4* positively regulates plant cell death by activating an oxidative burst.

Previously, it has been proved that PeREM6.5 interacts with PeRIN4 to enhance the enzymatic activity of PM H^+^-ATPase in *P. euphratica* [24]. In this study, a homologous gene, *CaRIN4-12*, of *PeRIN4* was cloned in pepper, and the interaction of CaREM1.4 and CaRIN4-12 was tested by Y2H, LUC, BIFC, and Co-IP, which suggests that they might be involved in the interaction between pepper and *R. solanacearum*. RIN4 is targeted by multiple pathogenic effectors, and consequently guarded by different immune receptors [41]. Previous studies have suggested that RIN4 negatively regulates plant PTI and ETI responses [37, 38]. After *CaRIN4-12* silencing, the transcript abundance of *CaREM1.4* was significantly upregulated. Furthermore, the expression of immune-associated genes was significantly upregulated. These results suggest that *CaRIN4-12* was a negative regulator of *C. annuum* against *R. solanacearum* infection*,* which negatively regulated the expression of *CaREM1.4*. However, recent studies have shown that RIN4 may control exocytosis to confer plant immunity. For example, EXO70 protein promotes callose deposition in the cell wall in the presence of flg22, which means RIN4 plays a positive role in plant basal defense [43]. The LUC assay showed that the other four RIN4s in pepper could also interact with CaREM1.4, but whether they play a negative regulatory role in the interaction with CaREM1.4 remains to be further studied.

Previous studies have shown that the defense-related genes play an important role in transgenic plants overexpressing *REM* genes. For example, the expression levels of defense-related genes in *ZmREM1.3*-overexpressing plants are significantly higher than those in control plants in response to *P. polysora* infection [20]. Moreover, transient overexpression of *NbREM4* in leaves of *N. benthamiana* induces expression of immunity-related genes [61]. In this study, we found that defense-related genes were significantly reduced and the expression level of the *CaRIN4-12* gene remained unchanged after the silencing of *CaREM1.4*, suggesting that the silencing of *CaREM1.4* weakened the resistance of pepper to *R. solanacearum*. Meanwhile, co-injection of *CaREM1.4* and *CaRIN4-12* in pepper led *CaRIN4-12* to inhibit *CaREM1.4-*induced necrosis. CaRIN4-12 interacted with CaREM1.4 and negatively regulated the pepper immune response to *R. solanacearum,* which was similar to the previous results*.*

**Figure 8:**
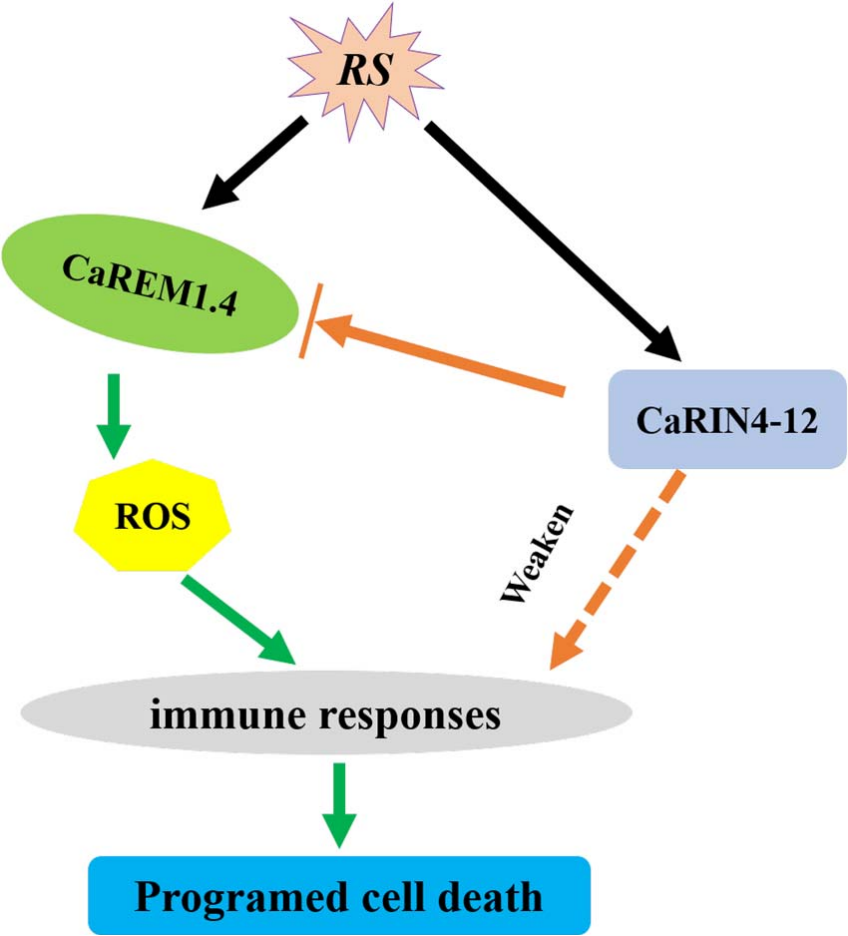
Proposed model for *CaREM1.4*- and *CaRIN4-12*-mediated immune response in pepper. In *R. solanacearum*-inoculated pepper leaves, *CaREM1.4* induces an immune response by stimulating ROS to produce cell death. However, CaRIN4-12, as a negative regulator of plant immunity, can interact with CaREM1.4 to decrease the production of ROS and cell death. *RS*, *R. solanacearum*.

In summary, we ascertained that *CaREM1.4* overexpression stimulated cell death in *C. annuum* and *N. benthamiana* leaves, and *CaREM1.4* knockdown decreased the resistance of pepper leaves to *R. solanacearum*. In addition, CaREM1.4 could interact with CaRIN4-12 and co-localize at the PM and cell nucleus. Furthermore, the *CaRIN4-12* gene inhibited the necrosis induced by the *CaREM1.4* gene. Our results suggested that *CaREM1.4* played a positive role in the plant ROS burst, cell death, and pathogenesis-related genes inducing resistance, while CaRIN4-12 played a negative regulatory role in these processes (Fig. 8). Our findings provided new evidence for comprehending the molecular regulatory network of plant cell death.

## Materials and methods

### Plant materials and *R. solanacearum* inoculation

Seeds of tobacco (*N. benthamiana*) and pepper (*C. annuum* cultivar ‘Zhong Jiao’，which exhibited medium resistance to *R. solanacearum*) were obtained from the Institute of Vegetables and Flowers, Chinese Academy of Agricultural Sciences (IVF CAAS). The growth conditions of these plant materials were as described in previous studies [62, 63]. The highly virulent *R. solanacearum* strain Px1 (phylotype I) used in this study was isolated from wilted samples of pepper from Shanxi province (China) and obtained from the IVF CAAS. Only one strain was used in this study. It was cultivated according to a method described previously [64, 65]. The strains were cultured overnight at 180 rpm, 28°C, suspended in 10 mM MgCl_2_ solution, and diluted to a final concentration of 10^8^ CFU/ml (OD_600_ = 0.8). For root inoculation, pepper plants at the eight-leaf stage were irrigated with *R. solanacearum* suspension. For leaf inoculation, the third leaf of pepper was inoculated with *R. solanacearum* using a 1-ml syringe without a needle, and the mock was inoculated with 10 mM MgCl_2_. Leaves were collected at the indicated time points for further analysis. The disease index (from 0 to 4) was calculated according to the wilting degree of pepper plants, namely 0 (no wilting), 1 (1–25% wilted), 2 (26–50% wilted), 3 (51–75% wilted), and 4 (76–100% wilted or dead) [58].

### Vector construction

To construct the vector for the transient expression assay, the ORF of *CaREM1.4* or *CaRIN4-12* without the termination codon was inserted into the SpeI restriction site of the pGinGFP2 vector with a GFP tag. To construct gene-silencing vectors for VIGS, *CaREM1.4* and *CaRIN4-12* gene fragments of 200–300 bp were identified by searching for the fragment with the lowest similarity to other pepper gene sequences on the NCBI database, then cloned into the gene-silencing vector pTRV2 (Invitrogen), separately. To construct the vector for the Y2H assay, the *CaREM1.4* ORF was constructed into the pGBKT7 and the ORF of *CaRIN4-12* was subcloned into pGADT7 with the EcoRI/BamHI sites. To construct the vector for Split-LUC assays, double digestion of nLUC and cLUC plasmids was performed with restriction enzymes BamHI/SalI. The ORF of *CaREM1.4* and *CaRIN4-12* was constructed into the cLUC and nLUC vector, respectively. To generate constructs for the BIFC assay, CaREM1.4 and CaRIN4-12 were fused to pSPYNE(R)173 and pSPYCE(M) vectors with the BamHI/XhoI sites, respectively [66]. To construct the vector for the Co-IP assay, the ORF sequence of *CaREM1.4* was constructed into the SpeI restriction site of pBI121MCS-mCherry vector. The primer sequences for construction of all vectors are listed in Supplementary Data Table S4.

### Gene identification and phylogenetic tree analysis

The *C. annuum* genome and protein sequences were downloaded with default parameters from the Ensembl Plants database [67]. To identify the *REM* gene family members of pepper plants, the *REM* gene sequences of *A. thaliana* and *S. lycopersicum* were used as query objects and BLASTP was used to perform multi-database search and comparison. REM proteins were confirmed to contain conserved REM domains and divided into different groups according to the description of Raffaele *et al*. [1]. To identify the RIN4-12 gene family members of pepper plants, the *C. annuum* UCD10Xv1.1 in the NCBI database was used as a reference genome.

Amino acid sequences of REM proteins from *C. annuum*, *A. thaliana*, *S. lycopersicum*, and *O. sativa* were collected from the Arabidopsis Information Resource, Sol Genomics Network, Rice Genome Annotation Project, and Ensembl database [67], respectively. Amino acid sequences of RIN4-12 proteins from *C. annuum* and *A. thaliana* were collected from the Ensembl database [67] and the Arabidopsis Information Resource. Accession numbers of all REMs and RIN4-12 are listed in Supplementary Data Table S1. ClustalX and MEGA7 software was used for sequence alignment and phylogenetic tree construction [68].

### Gene structure and promoter analysis

Genomic sequences and ORFs of CaREMs were acquired from the Ensembl database [67]. The exon and intron structures were identified with Gene Structure Display Server version 2.0 [69]. The MEME program [70] was used to analyze conserved motifs of *CaREM* genes and DNAMAN software was used to align amino acid sequences. The chromosomal locations of *CaREM* genes were drawn by BTool. The ExPASy tools were used to predict some biochemical properties, such as isoelectric point and molecular weight. Presumed *cis*-acting regulatory DNA elements of *CaREM* genes were identified in the 2.0-kb upstream region preceding the translation start site as described previously [71, 72]. The putative stress or hormone-responsive *cis*-acting regulatory elements in these sequences were analyzed as in previous studies [73].

### RNA extraction and qRT–PCR analysis

Total RNA extraction of plants and qRT–PCR analysis were performed as detailed in our previous studies [74, 75]; three biological replications of each experiment were prepared. A qPCR System (Bio-Rad, USA) was used to quantify the expression of genes with *CaActin* (GQ339766) as internal control. Livak and Schmittgen’s method was used for data analysis [76]. qRT–PCR primers are listed in Supplementary Data Table S4. The major raw qPCR data are given in Supplementary Data Table S5.

### Virus-induced gene silencing assay

A TRV-based VIGS assay was used to transiently silence *CaREM1.4* and *CaRIN4-12* in this study. The procedure was performed as previously described [63, 74]. To avoid the possible silencing of other homologues, a specific fragment of *CaREM1.4* and *CaRIN4-12* was amplified from the pepper cDNA library using gene-specific primers (Supplementary Data Table S4) and cloned into the VIGS vector pTRV2. *A. tumefaciens* strains with pTRV1 vector were respectively mixed with constructs containing pTRV2:*CaREM1.4*, pTRV2:*CaRIN4–12*, pTRV2:*00*, and pTRV2:*CaPDS* at a 1:1 ratio. The mixed *A. tumefaciens* cells were infiltrated into 12-day-old pepper plants. qRT–PCR was used to detect the silencing efficiency of plants after *R. solanacearum* infection.

### Determination of *R. solanacearum* colony-forming units

Pepper leaves injected with *R. solanacearum* were punched and sampled at 48 hpi with a 1-cm hole punch. Leaves from each plant were punched with six holes and put into a 2-ml centrifuge tube; 400 μl of ddH_2_O was added and the samples were mashed with a sterilized grinding rod. Sterile water (600 μl) was added and the samples were mixed well, followed by dilution to 10^−4^, 10^−5^, and 10^−6^ gradients. Finally, 400 μl of each gradient dilution was spread evenly on TTC solid medium and cultured in an incubator at 28°C. After 2 days, the number of colonies on the plate was counted using ImageJ software (National Institutes of Health). Previous studies report more details on this process [64, 65].

### Transient expression of *CaREM1.4* or *CaRIN4-12* in pepper plants

For the transient expression assay, *A. tumefaciens* suspensions (OD_600_ = 0.6) containing the pBinGFP2-CaREM1.4 and pBinGFP2-CaRIN4-12 constructs were injected into pepper leaves, respectively. pBinGFP2 was used as a control. At different time points after infiltration, we harvested the injected pepper leaves for subsequent use.

### Histochemical staining, electrolyte leakage and reactive oxygen species accumulation determination

The leaves were histologically stained with DAB for H_2_O_2_ measurement and trypan blue staining for cell necrosis assays according to a previously published method [32]. Ionic conductivity was used to quantitatively detect cell death in pepper leaves as described previously [77]. Six leaf disks with a diameter of 1 cm were taken from the injection area of the leaves and, after soaking in 5 ml ddH_2_O for 5 hours, were measured as value A using a conductivity meter (Mettler-Toledo, China). After boiling the centrifuge tube with the leaf disks for 20 minutes, ion conductivity was measured as value B and the ion leakage was calculated as (value A/value B) × 100. H_2_O_2_ is one of the important reactive oxygen species. The ROS contents of plant leaves were quantified using Micro Hydrogen Peroxide (H_2_O_2_) Assay Kit (Solarbio, China) with reference to the instructions.

### Protein interaction assays

For the Y2H analysis, pGBKT7-CaREM1.4 and pGADT7-CaRIN4-12 were co-transferred into the AH109 strains using the LiAc (lithium acetate) method. Plasmids pGBKT7-P53/pGADT7-SV40 and pGBKT7-CaREM1.4/pGADT7 were transformed into yeast strains as positive and negative controls, respectively. Yeast colonies grown on SD medium lacking Trp/Leu/His/Ade were identified as positive clones. X-*α*-gal was used to detect interactions by the development of a blue color. Split-LUC analysis was performed as previously described [78]. The vectors CaREM1.4-nLUC and CaRIN4-12-cLUC were co-injected into tobacco leaves. Luciferin (1 mM; AbMole) was applied to the injected area at 48 hpi and LUC activity was detected by the PlantView100 system. For the BIFC assay, CaREM1.4-nYFP and CaRIN4-12-cYFP constructs containing yellow fluorescent protein (YFP) were co-expressed in *N. benthamiana* leaves. The control group was a combination of CaREM1.4-nYFP and cYFP. YFP fluorescence signals were imaged using a confocal microscope. For the Co-IP assay, pBI121MCS-CaREM1.4/pBinGFP2-CaRIN4-12 and pBI121MCS-CaREM1.4/pBinGFP2 (as a negative control) were co-expressed in tobacco leaves, separately. After 48 hpi, the injected leaves were clipped and ground, and homogenized in RIPA buffer. The extracts were centrifuged and then the supernatant was aspirated. GFP-TRAP beads were added to the supernatant and incubated for 2 hours. The purified samples were then boiled for western blot analysis. Anti-GFP and anti-mCherry were used to detect proteins.

### Co-localization of CaREM1.4 and CaRIN4-12

To verify the co-localization of CaREM1.4 and CaRIN4-12 in *N. benthamiana*, *A. tumefaciens* carrying pBI121MCS:CaREM1.4 and pCAMBIA1302:CaRIN4-12 vectors were co-injected into *N*. *benthamiana* leaves [79]. Tawpi6-mCherry, a plasma membrane marker protein, served as a positive control [80]. GFP and mCherry fluorescence signals were imaged using an Olympus FV3000 microscope with a wavelength of 488 and 620 nm.

### Statistical analysis

The means ± standard deviations of data were analyzed by Microsoft Excel software and significant differences between control and experimental groups were calculated by Student’s *t* test using GraphPad Prism 8.0.

## Acknowledgements

We are grateful to the Natural Science Basic Research Program of Shaanxi Province-Key Project (2023-JC-ZD-12), the National Key R&D Program of China (2021YFD1401000), the Shaanxi Innovation Team Project (2018TD-004), and the 111 Project of the Ministry of Education of China (B07049) for their support of this study. We appreciate the advice of Professor Peng Chen and Minghui Lu (Northwest A&F University). We thank Zelong Li (Northwest A&F University) for improving the phylogenetic tree, Dr Hua Zhao and Dr Fengping Yuan (State Key Laboratory of Crop Stress Biology) for their support in confocal microscope.

## Author contributions

Z.S.K., X.J.W., and X.M.Z. conceived the framework of the paper; G.G. and P.F.G. contributed original ideas. Y.Q.Z., S.Y.G., M.L., F.Z., C.W., and H.K.L. performed the experiments; Y.Q.Z. analyzed the data. X.M.Z. and Y.Q.Z. wrote the article.

## Data availability

The Ensembl Plants database was used to search the genome sequence data used in this paper, with accession numbers as follows: *CaREM1.4* (PHT78353) and *CaRIN4-12* (PHT66856).

## Conflict of interest statement

The authors declare no conflicting of interest.

## Supplementary Data

Supplementary data is available at *Horticulture Research* online.

## Supplementary Material

Web_Material_uhad053

## References

[1] Raffaele S , Mongrand S, Gamas P et al. Genome-wide annotation of remorins, a plant-specific protein family: evolutionary and functional perspectives. Plant Physiol. 2007;145:593–600. 17984200 10.1104/pp.107.108639 PMC2048807

[2] Gui J , Zheng S, Liu C et al. OsREM4.1 interacts with OsSERK1 to coordinate the interlinking between abscisic acid and brassinosteroid signaling in rice. Dev Cell. 2016;38:201–13. 27424498 10.1016/j.devcel.2016.06.011

[3] Farmer EE , Pearce G, Ryan CA. In vitro phosphorylation of plant plasma membrane proteins in response to the proteinase inhibitor inducing factor. Proc Natl Acad Sci USA. 1989;86:1539–42. 16578842 10.1073/pnas.86.5.1539 PMC286733

[4] Jacinto T , Farmer EE, Ryan CA. Purification of potato leaf plasma membrane protein pp34, a protein phosphorylated in response to oligogalacturonide signals for defense and development. Plant Physiol. 1993;103:1393–7. 12232033 10.1104/pp.103.4.1393 PMC159131

[5] Yue J , Li C, Liu Y et al. A remorin gene *SiREM6*, the target gene of SiARDP, from foxtail millet (*Setaria italica*) promotes high salt tolerance in transgenic *Arabidopsis*. PLoS One. 2014;9:e100772. 24967625 10.1371/journal.pone.0100772 PMC4072699

[6] Badawi MA , Agharbaoui Z, Zayed M et al. Genome-wide identification and characterization of the wheat remorin (*Ta*REM) family during cold acclimation. Plant Genome. 2019;12:180040. 10.3835/plantgenome2018.06.0040 PMC12810125 31290927

[7] Martinez D , Legrand A, Gronnier J et al. Coiled-coil oligomerization controls localization of the plasma membrane REMORINs. J Struct Biol. 2019;206:12–9. 29481850 10.1016/j.jsb.2018.02.003

[8] Perraki A , Cacas JL, Crowet JM et al. Plasma membrane localization of *Solanum tuberosum* remorin from group 1, homolog 3 is mediated by conformational changes in a novel C-terminal anchor and required for the restriction of potato virus X movement. Plant Physiol. 2012;160:624–37. 22855937 10.1104/pp.112.200519 PMC3461544

[9] Checker VG , Khurana P. Molecular and functional characterization of mulberry EST encoding remorin (MiREM) involved in abiotic stress. Plant Cell Rep. 2013;32:1729–41. 23942844 10.1007/s00299-013-1483-5

[10] Cai J , Qin G, Chen T et al. The mode of action of remorin1 in regulating fruit ripening at transcriptional and post-transcriptional levels. New Phytol. 2018;219:1406–20. 29978907 10.1111/nph.15264

[11] Gouguet P , Gronnier J, Legrand A et al. Connecting the dots: from nanodomains to physiological functions of REMORINs. Plant Physiol. 2021;185:632–49. 33793872 10.1093/plphys/kiaa063 PMC8133660

[12] Raffaele S , Bayer E, Lafarge D et al. Remorin, a Solanaceae protein resident in membrane rafts and plasmodesmata, impairs *Potato virus X* movement. Plant Cell. 2009;21:1541–55. 19470590 10.1105/tpc.108.064279 PMC2700541

[13] Lefebvre B , Timmers T, Mbengue M et al. A remorin protein interacts with symbiotic receptors and regulates bacterial infection. Proc Natl Acad Sci USA. 2010;107:2343–8. 20133878 10.1073/pnas.0913320107 PMC2836688

[14] Perraki A , Binaghi M, Mecchia MA et al. StRemorin1.3 hampers potato virus X TGBp1 ability to increase plasmodesmata permeability, but does not interfere with its silencing suppressor activity. FEBS Lett. 2014;588:1699–705. 24657438 10.1016/j.febslet.2014.03.014

[15] Kong CY , Luo YP, Duan TT et al. Potato remorin gene StREMa4 cloning and its spatiotemporal expression pattern under *Ralstonia solanacearum* and plant hormones treatment. Phytoparasitica. 2016;44:575–84.

[16] Reymond P , Kunz B, Paul-Pletzer K et al. Cloning of a cDNA encoding a plasma membrane-associated, uronide binding phosphoprotein with physical properties similar to viral movement proteins. Plant Cell. 1996;8:2265–76. 8989883 10.1105/tpc.8.12.2265 PMC161351

[17] Benschop JJ , Mohammed S, O'Flaherty M et al. Quantitative phosphoproteomics of early elicitor signaling in *Arabidopsis*. Mol Cell Proteomics. 2007;6:1198–214. 17317660 10.1074/mcp.M600429-MCP200

[18] Jarsch IK , Ott T. Perspectives on remorin proteins, membrane rafts, and their role during plant-microbe interactions. Mol Plant-Microbe Interact. 2011;24:7–12. 21138374 10.1094/MPMI-07-10-0166

[19] Liu J , Elmore JM, Coaker G. Investigating the functions of the RIN4 protein complex during plant innate immune responses. Plant Signal Behav. 2009;4:1107–10. 20514222 10.4161/psb.4.12.9944 PMC2819432

[20] Wang S , Chen Z, Tian L et al. Comparative proteomics combined with analyses of transgenic plants reveal ZmREM1.3 mediates maize resistance to southern corn rust. Plant Biotechnol J. 2019;17:2153–68. 30972847 10.1111/pbi.13129 PMC6790363

[21] Jamann TM , Luo X, Morales L et al. A remorin gene is implicated in quantitative disease resistance in maize. Theor Appl Genet. 2016;129:591–602. 26849237 10.1007/s00122-015-2650-6

[22] Son S , Oh C, An C. *Arabidopsis thaliana* remorins interact with SnRK1 and play a role in susceptibility to beet curly top virus and beet severe curly top virus. Plant Pathol J. 2014;30:269–78. 25289013 10.5423/PPJ.OA.06.2014.0061 PMC4181108

[23] Bozkurt TO , Richardson A, Dagdas YF et al. The plant membrane-associated REMORIN1.3 accumulates in discrete perihaustorial domains and enhances susceptibility to *Phytophthora infestans*. Plant Physiol. 2014;165:1005–18. 24808104 10.1104/pp.114.235804 PMC4081318

[24] Zhang H , Deng C, Wu X et al. *Populus euphratica* remorin 6.5 activates plasma membrane H^+^-ATPases to mediate salt tolerance. Tree Physiol. 2020;40:731–45. 32159803 10.1093/treephys/tpaa022

[25] Lam E . Controlled cell death, plant survival and development. Nat Rev Mol Cell Biol. 2004;5:305–15. 15071555 10.1038/nrm1358

[26] Fuchs Y , Steller H. Programmed cell death in animal development and disease. Cell. 2011;147:742–58. 22078876 10.1016/j.cell.2011.10.033 PMC4511103

[27] Singh Y , Nair AM, Verma PK. Surviving the odds: from perception to survival of fungal phytopathogens under host-generated oxidative burst. Plant Commun. 2021;2:100142. 34027389 10.1016/j.xplc.2021.100142 PMC8132124

[28] Kaneda T , Taga Y, Takai R et al. The transcription factor OsNAC4 is a key positive regulator of plant hypersensitive cell death. EMBO J. 2009;28:926–36. 19229294 10.1038/emboj.2009.39 PMC2670867

[29] van Breusegem F , Dat JF. Reactive oxygen species in plant cell death. Plant Physiol. 2006;141:384–90. 16760492 10.1104/pp.106.078295 PMC1475453

[30] Qin G , Meng X, Wang Q et al. Oxidative damage of mitochondrial proteins contributes to fruit senescence: a redox proteomics analysis. J Proteome Res. 2009;8:2449–62. 19239264 10.1021/pr801046m

[31] Tian S , Qin G, Li B. Reactive oxygen species involved in regulating fruit senescence and fungal pathogenicity. Plant Mol Biol. 2013;82:593–602. 23515879 10.1007/s11103-013-0035-2

[32] Cai J , Chen T, Wang Y et al. SlREM1 triggers cell death by activating an oxidative burst and other regulators. Plant Physiol. 2020;183:717–32. 32317359 10.1104/pp.20.00120 PMC7271787

[33] Lam E , Kato N, Lawton M. Programmed cell death, mitochondria and the plant hypersensitive response. Nature. 2001;411:848–53. 11459068 10.1038/35081184

[34] Suzuki N , Miller G, Morales J et al. Respiratory burst oxidases: the engines of ROS signaling. Curr Opin Plant Biol. 2011;14:691–9. 21862390 10.1016/j.pbi.2011.07.014

[35] Yoshioka H , Numata N, Nakajima K et al. *Nicotiana benthamiana* gp91phox homologs NbrbohA and NbrbohB participate in H_2_O_2_ accumulation and resistance to *Phytophthora infestans*. Plant Cell. 2003;15:706–18. 12615943 10.1105/tpc.008680 PMC150024

[36] Yoshie Y , Goto K, Takai R et al. Function of the rice gp91phox homologs *OsrbohA* and *OsrbohE* genes in ROS-dependent plant immune responses. Plant Biotechnol J. 2005;22:127–35.

[37] Zhao G , Guo D, Wang L et al. Functions of RPM1-interacting protein 4 in plant immunity. Planta. 2021;253:11. 33389186 10.1007/s00425-020-03527-7

[38] Belkhadir Y , Nimchuk Z, Hubert DA et al. *Arabidopsis* RIN4 negatively regulates disease resistance mediated by RPS2 and RPM1 downstream or independent of the NDR1 signal modulator and is not required for the virulence functions of bacterial type III effectors AvrRpt2 or AvrRpm1. Plant Cell. 2004;16:2822–35. 15361584 10.1105/tpc.104.024117 PMC520974

[39] Kim MG , da Cunha L, McFall AJ et al. Two *Pseudomonas syringae* type III effectors inhibit RIN4-regulated basal defense in *Arabidopsis*. Cell. 2005;121:749–59. 15935761 10.1016/j.cell.2005.03.025

[40] Selote D , Kachroo A. RIN4-like proteins mediate resistance protein-derived soybean defense against *Pseudomonas syringae*. Plant Signal Behav. 2010;5:1453–6. 21051954 10.4161/psb.5.11.13462 PMC3115253

[41] Axtell MJ , Staskawicz BJ. Initiation of RPS2-specified disease resistance in *Arabidopsis* is coupled to the AvrRpt2-directed elimination of RIN4. Cell. 2003;112:369–77. 12581526 10.1016/s0092-8674(03)00036-9

[42] Day B , Dahlbeck D, Huang J et al. Molecular basis for the RIN4 negative regulation of RPS2 disease resistance. Plant Cell. 2005;17:1292–305. 15749765 10.1105/tpc.104.030163 PMC1088003

[43] Wu X , Huang J, Cao Y et al. The resistance associated protein RIN4 promotes the extracellular transport of AtEXO70E2. Biochem Biophys Res Commun. 2021;555:40–5. 33812057 10.1016/j.bbrc.2021.03.072

[44] Lebeau A , Daunay MC, Frary A et al. Bacterial wilt resistance in tomato, pepper, and eggplant: genetic resources respond to diverse strains in the *Ralstonia solanacearum* species complex. Phytopathology. 2011;101:154–65. 20795852 10.1094/PHYTO-02-10-0048

[45] Noman A , Liu Z, Yang S et al. Expression and functional evaluation of CaZNF830 during pepper response to *Ralstonia solanacearum* or high temperature and humidity. Microb Pathog. 2018;118:336–46. 29614367 10.1016/j.micpath.2018.03.044

[46] Zander M , Chen S, Imkampe J et al. Repression of the *Arabidopsis thaliana* jasmonic acid/ethylene-induced defense pathway by TGA-interacting glutaredoxins depends on their C-terminal ALWL motif. Mol Plant. 2012;5:831–40. 22207719 10.1093/mp/ssr113

[47] Rabara RC , Tripathi P, Lin J et al. Dehydration-induced WRKY genes from tobacco and soybean respond to jasmonic acid treatments in BY-2 cell culture. Biochem Biophys Res Commun. 2013;431:409–14. 23333328 10.1016/j.bbrc.2012.12.156

[48] Jung HW , Kim KD, Hwang BK. Identification of pathogen-responsive regions in the promoter of a pepper lipid transfer protein gene (CALTPI) and the enhanced resistance of the CALTPI transgenic *Arabidopsis* against pathogen and environmental stresses. Planta. 2005;221:361–73. 15654638 10.1007/s00425-004-1461-9

[49] Rocher A , Dumas C, Cock JM. A W-box is required for full expression of the SA-responsive gene *SFR2*. Gene. 2005;344:181–92. 15656984 10.1016/j.gene.2004.09.016

[50] Luo Y , Caldwell KS, Wroblewski T et al. Proteolysis of a negative regulator of innate immunity is dependent on resistance genes in tomato and *Nicotiana benthamiana* and induced by multiple bacterial effectors. Plant Cell. 2009;21:2458–72. 19671880 10.1105/tpc.107.056044 PMC2751963

[51] Ray SK , Macoy DM, Kim WY et al. Role of RIN4 in regulating PAMP-triggered immunity and effector-triggered immunity: current status and future perspectives. Mol Cells. 2019;42:503–11. 31362467 10.14348/molcells.2019.2433 PMC6681865

[52] Konrad SS , Popp C, Stratil TF et al. S-acylation anchors remorin proteins to the plasma membrane but does not primarily determine their localization in membrane microdomains. New Phytol. 2014;203:758–69. 24897938 10.1111/nph.12867

[53] Marin M , Ott T. Phosphorylation of intrinsically disordered regions in remorin proteins. Front Plant Sci. 2012;3:86. 22639670 10.3389/fpls.2012.00086 PMC3355724

[54] Gui J , Zheng S, Shen J et al. Grain setting defect1 (GSD1) function in rice depends on S-acylation and interacts with actin 1 (OsACT1) at its C-terminal. Front Plant Sci. 2015;6:804. 26483819 10.3389/fpls.2015.00804 PMC4590517

[55] Lamb C , Dixon RA. The oxidative burst in plant disease resistance. Annu Rev Plant Physiol Plant Mol Biol. 1997;48:251–75. 15012264 10.1146/annurev.arplant.48.1.251

[56] Morel JB , Dangl JL. The hypersensitive response and the induction of cell death in plants. Cell Death Differ. 1997;4:671–83. 16465279 10.1038/sj.cdd.4400309

[57] Greenberg JT , Yao N. The role and regulation of programmed cell death in plant-pathogen interactions. Cell Microbiol. 2004;6:201–11. 14764104 10.1111/j.1462-5822.2004.00361.x

[58] Overmyer K , Brosché M, Kangasjärvi J. Reactive oxygen species and hormonal control of cell death. Trends Plant Sci. 2003;8:335–42. 12878018 10.1016/S1360-1385(03)00135-3

[59] Jacobson MD . Reactive oxygen species and programmed cell death. Trends Biochem Sci. 1996;21:83–6. 8882579

[60] Torres MA . ROS in biotic interactions. Physiol Plant. 2010;138:414–29. 20002601 10.1111/j.1399-3054.2009.01326.x

[61] Albers P , Üstün S, Witzel K et al. A remorin from *Nicotiana benthamiana* interacts with the *Pseudomonas* type-III effector protein HopZ1a and is phosphorylated by the immune-related kinase PBS1. Mol Plant Microbe Interact. 2019;32:1229–42. 31012804 10.1094/MPMI-04-19-0105-R

[62] Choi DS , Hwang IS, Hwang BK. Requirement of the cytosolic interaction between PATHOGENESIS-RELATED PROTEIN10 and LEUCINE-RICH REPEAT PROTEIN1 for cell death and defense signaling in pepper. Plant Cell. 2012;24:1675–90. 22492811 10.1105/tpc.112.095869 PMC3398571

[63] Dang F , Wang Y, She J et al. Overexpression of CaWRKY27, a subgroup IIe WRKY transcription factor of *Capsicum annuum*, positively regulates tobacco resistance to *Ralstonia solanacearum* infection. Physiol Plant. 2014;150:397–411. 24032447 10.1111/ppl.12093

[64] Yang S , Shi Y, Zou L et al. Pepper CaMLO6 negatively regulates *Ralstonia solanacearum* resistance and positively regulates high temperature and high humidity responses. Plant Cell Physiol. 2020;61:1223–38. 32343804 10.1093/pcp/pcaa052

[65] Shen L , Yang S, Yang F et al. CaCBL1 acts as a positive regulator in pepper response to *Ralstonia solanacearum*. Mol Plant Microbe Interact. 2020;33:945–57. 32209000 10.1094/MPMI-08-19-0241-R

[66] Waadt R , Schmidt LK, Lohse M et al. Multicolor bimolecular fluorescence complementation reveals simultaneous formation of alternative CBL/CIPK complexes in planta. Plant J. 2008;56:505–16. 18643980 10.1111/j.1365-313X.2008.03612.x

[67] Kersey PJ , Allen JE, Christensen M et al. Ensembl Genomes 2013: scaling up access to genome-wide data. Nucleic Acids Res. 2014;42:546–52. 10.1093/nar/gkt979 PMC3965094 24163254

[68] Kumar R , Mishra BK, Lahiri T et al. PCV: an alignment free method for finding homologous nucleotide sequences and its application in phylogenetic study. Interdiscip Sci. 2017;9:173–83. 26825665 10.1007/s12539-015-0136-5

[69] Hu B , Jin J, Guo AY et al. GSDS 2.0: an upgraded gene feature visualization server. Bioinformatics. 2015;31:1296–7. 25504850 10.1093/bioinformatics/btu817 PMC4393523

[70] Bailey TL , Elkan C. Fitting a mixture model by expectation maximization to discover motifs in biopolymers. Proc Int Conf Intell Syst Mol Biol. 1994;2:28–36. 7584402

[71] Badawi M , Reddy YV, Agharbaoui Z et al. Structure and functional analysis of wheat ICE (inducer of CBF expression) genes. Plant Cell Physiol. 2008;49:1237–49. 18635580 10.1093/pcp/pcn100

[72] Li Q , Byrns B, Badawi MA et al. Transcriptomic insights into phenological development and cold tolerance of wheat grown in the field. Plant Physiol. 2018;176:2376–94. 29259104 10.1104/pp.17.01311 PMC5841705

[73] Lescot M , Déhais P, Thijs G et al. PlantCARE, a database of plant cis-acting regulatory elements and a portal to tools for in silico analysis of promoter sequences. Nucleic Acids Res. 2002;30:325–7. 11752327 10.1093/nar/30.1.325 PMC99092

[74] Cai H , Yang S, Yan Y et al. CaWRKY6 transcriptionally activates CaWRKY40, regulates *Ralstonia solanacearum* resistance, and confers high-temperature and high-humidity tolerance in pepper. J Exp Bot. 2015;66:3163–74. 25873659 10.1093/jxb/erv125

[75] Zhang Q , Zhang X, Zhuang R et al. *TaRac6* is a potential susceptibility factor by regulating the ROS burst negatively in the wheat–*Puccinia striiformis* f. sp. *tritici* interaction. Front Plant Sci. 2020;11:716. 32695124 10.3389/fpls.2020.00716 PMC7338558

[76] Livak KJ , Schmittgen TD. Analysis of relative gene expression data using real-time quantitative PCR and the 2(-Delta Delta C(T)) method. Methods. 2001;25:402–8. 11846609 10.1006/meth.2001.1262

[77] Yu X , Tang J, Wang Q et al. The RxLR effector Avh241 from *Phytophthora sojae* requires plasma membrane localization to induce plant cell death. New Phytol. 2012;196:247–60. 22816601 10.1111/j.1469-8137.2012.04241.x

[78] Chen X , Liu X, Gao Y et al. Application of firefly luciferase (Luc) as a reporter gene for the chemoautotrophic and acidophilic *Acidithiobacillus* spp. Curr Microbiol. 2020;77:3724–30. 32945904 10.1007/s00284-020-02195-w

[79] Cheng Y , Wang X, Yao J et al. Characterization of protein kinase PsSRPKL, a novel pathogenicity factor in the wheat stripe rust fungus. Environ Microbiol. 2015;17:2601–17. 25407954 10.1111/1462-2920.12719

[80] Imai R , Koike M, Sutoh K et al. Molecular characterization of a cold-induced plasma membrane protein gene from wheat. Mol Gen Genomics. 2005;274:445–53. 10.1007/s00438-005-0050-3 16184390

