# Supplementary material for: CaREM1.4 interacts with CaRIN4 to regulate Ralstonia solanacearum tolerance by triggering cell death in pepper
Source: Hortic Res. 2023 Mar 28;10(5):uhad053. doi: 10.1093/hr/uhad053 (PMC10199716; doi:10.1093/hr/uhad053)
Supplement: Web_Material_uhad053 [file web_material_uhad053.zip › Supplemental Figure.docx]

**
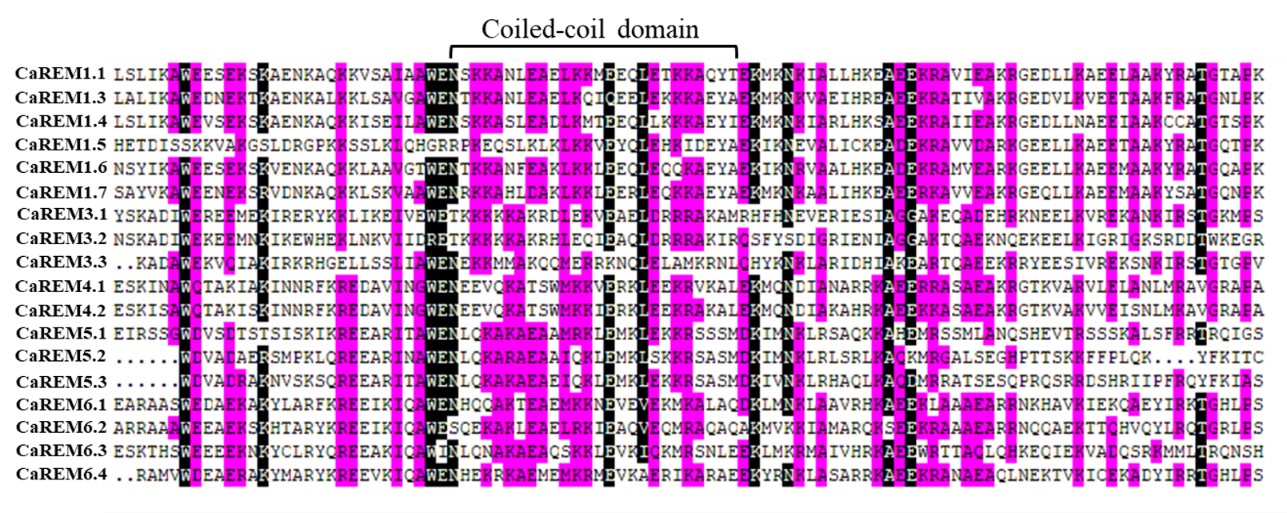
**

**Supplemental Fig S1. Multiple sequence alignment of 18 pepper remorin C-motifs.** The coiled-coil domain in the remorin C-motif is boxed. Conserved amino acids and blocks of similar amino acid residues are shaded in black and purple, respectively.

**
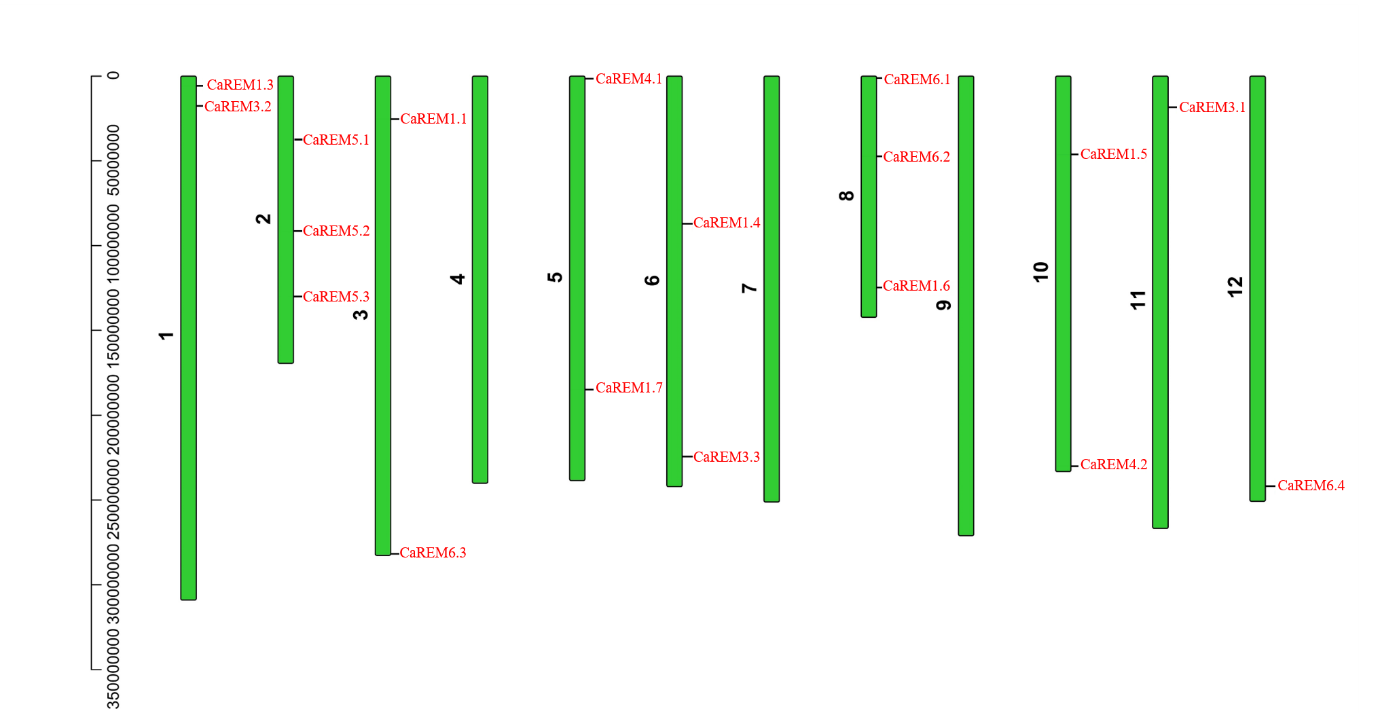
**

**Supplemental Fig S2. Chromosomal locations of *CaREM* genes in the pepper genome.** The distributions of the 18 remorin genes were determined according to the scaffold number and are shown in red.


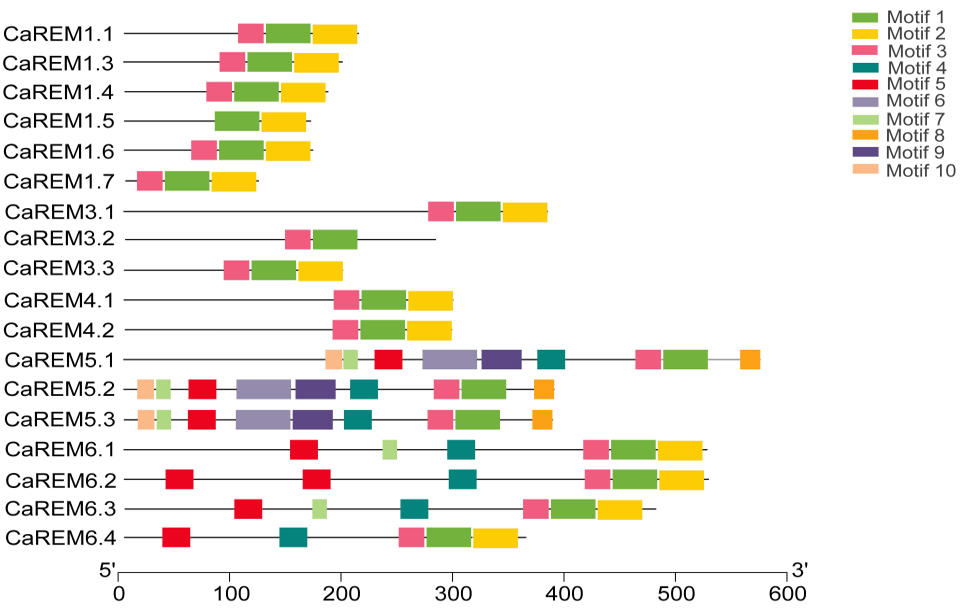


**Supplemental Fig S3. Schematic diagram of the conserved motifs in 18 pepper REM genes.** The motifs were identified by Multiple Em for Motif Elucidation (MEME) software. Each colored number represents a motif. The remorin C-domain represents Motif 1 and the remorin N-domain represents Motif 2. The other motifs are shown in different colors. The consensus sequences of the motifs identified by MEME and their function identified by the PROSITE and Eukaryotic Linear Motif resource tools are presented in Supplemental Table S2.


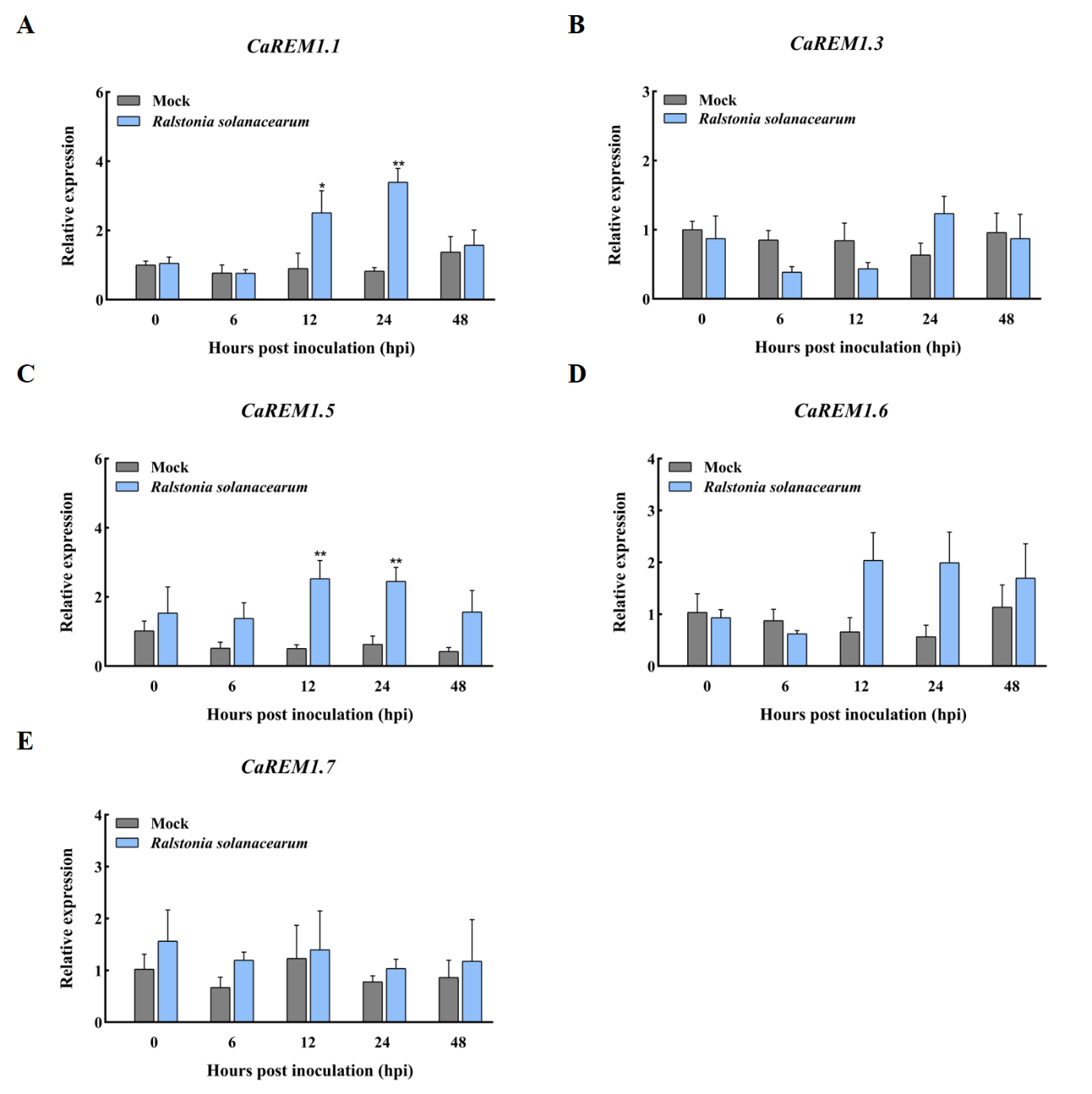


**Supplemental Fig S4. Transcript levels of *CaREM1.1* (A), *CaREM1.3* (B), *CaREM1.5* (C), *CaREM1.6* (D), *CaREM1.7* (E) in *R. solanacearum*-inoculated plants at 0, 6, 12, 24, and 48 hpi.** Untreated leaves act as the control. The relative expression of *CaREMs* was calculated using the comparative threshold method (2^–ΔΔCt^). Expression levels were normalized to *CaActin*. Values were derived from three biological repetitions. Asterisks indicate a significant difference (**P* < 0.05, ***P* < 0.01) compared to the untreated control according to Student’s *t* test.


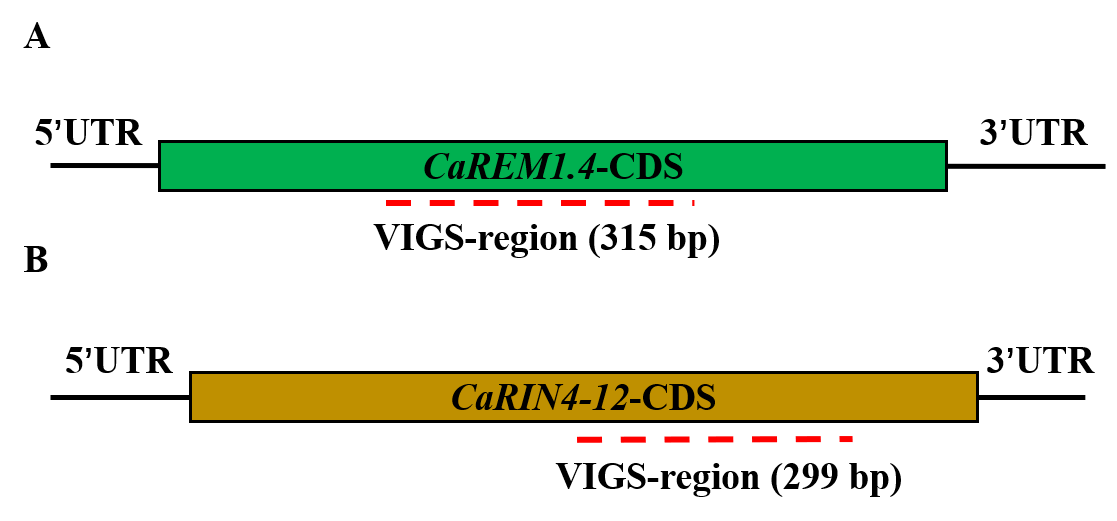


**Supplemental Fig S5.** **Schematic view of the pTRV2-VIGS construct design about *CaREM1.4* (A) and *CaRIN4-12* (B).**


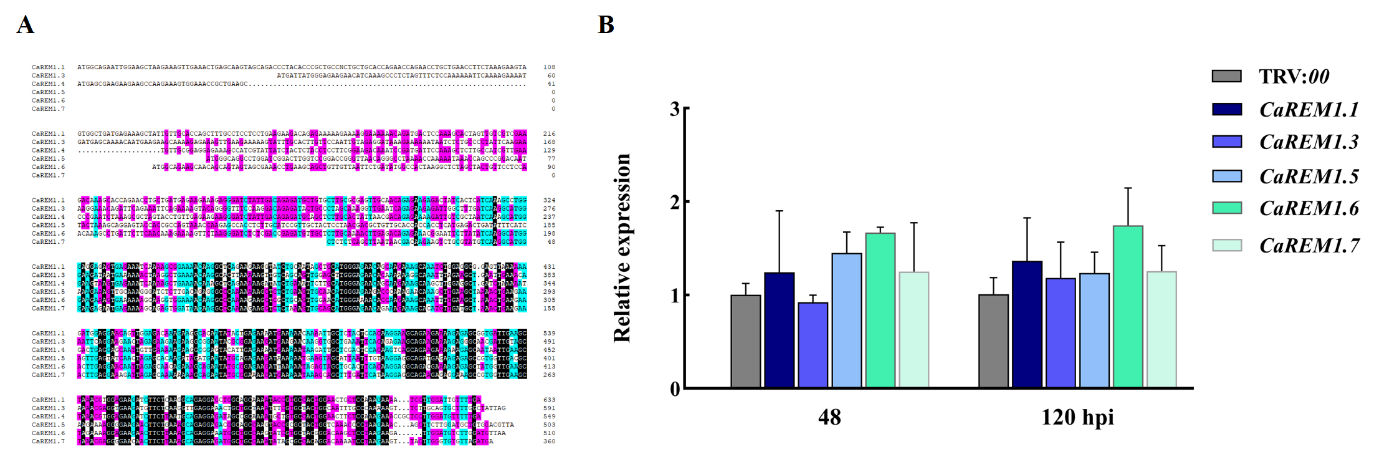


**Supplemental Fig S6. Nucleic acid sequences alignment of subgroup I CaREMs (A) and transcript levels of** **other subgroup I *CaREMs* in *CaREM1.4*-knockdown plants (B).** Expression profiles of other subgroup I *CaREMs* were assessed in *CaREM1.4*-knockdown plants compared with control plants. The data were normalized to the *CaActin* gene. Three independent biological replications were performed.

**
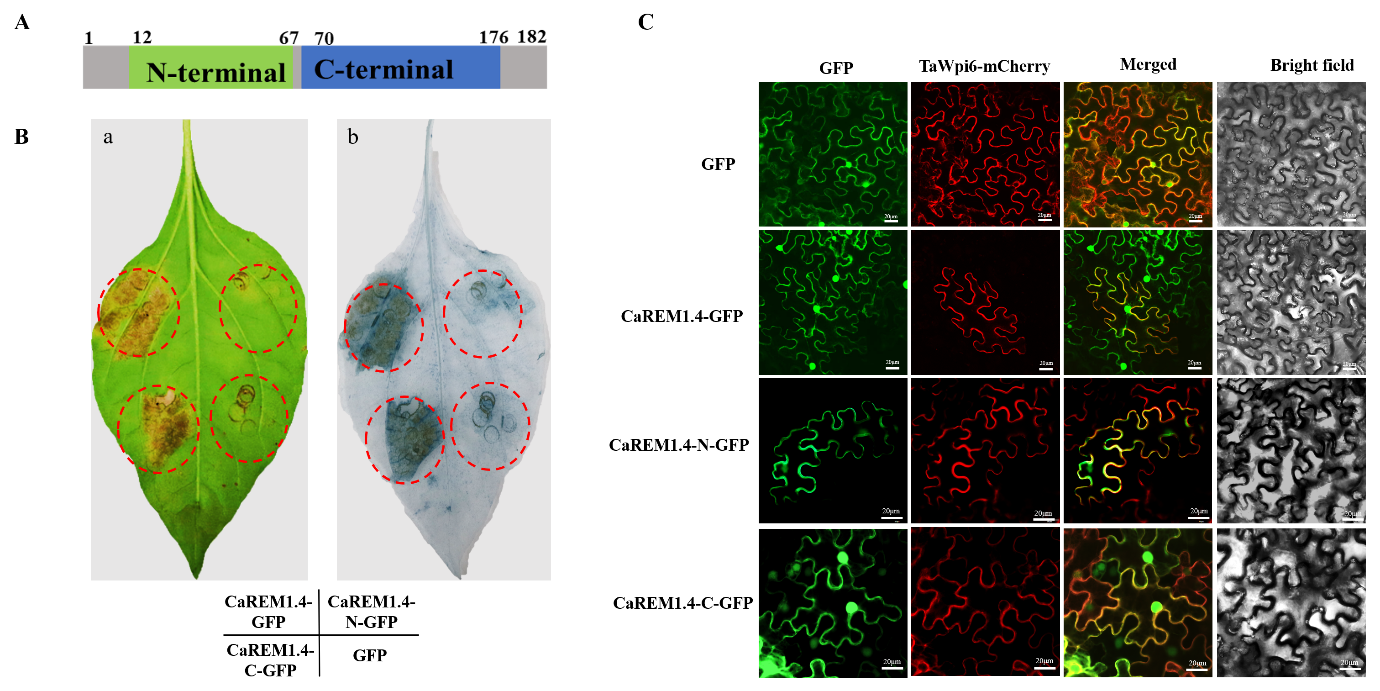
**

**Supplemental Fig S7. The C-terminal domain of CaREM1.4 is the key domain that causes cell death in pepper leaves.** A, The amino acid sequence of CaREM1.4 was predicted with two domains from N-terminus to C-terminus. Domains were detected by SMART (http://smart.embl-heidelberg.de/). B, Pepper leaves were infiltrated with *A. tumefaciens* GV3101 carrying CaREM1.4-GFP, CaREM1.4-N-GFP, CaREM1.4-C-GFP and GFP (empty vector). Phenotypes of infiltrated pepper leaves at 4 days after *Agroinfiltration*, by trypan blue staining. The red circles indicate the infiltration areas. C, Subcellular localization of CaREM1.4-GFP, CaREM1.4-N-GFP and CaREM1.4-C-GFP in *N. benthamiana* leaves and the fluorescence was observed at 48 hpi. GFP fluorescence is in green; TaWpi6-mCherry (red fluorescence) indicates TaWpi6 labeling the plasma membrane. Bar = 20 μm.

**
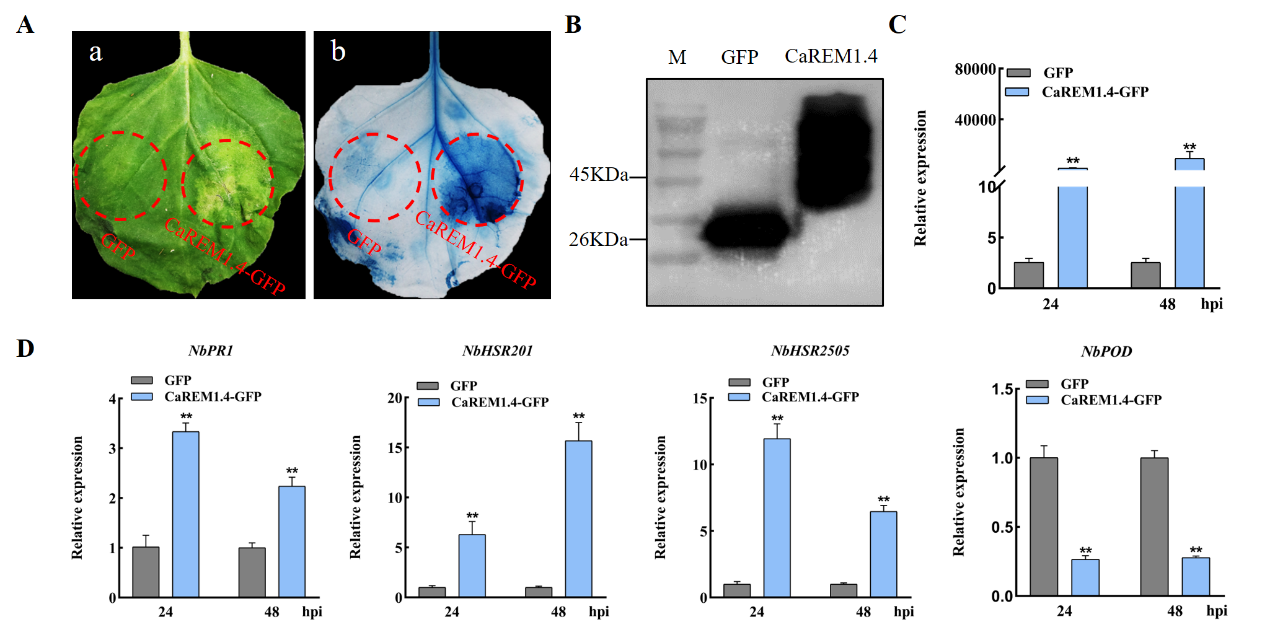
**

**Supplemental Fig S8. The effect of transient overexpression of *CaREM1.4* on the hypersensitive response (HR) mimic cell death and the expression of immunity-associated genes in *N. benthamiana* leaves.** A: Leaves of *N. benthamiana* were infiltrated with *A. tumefaciens* carrying CaREM1.4-GFP (on the left half) or pBinGFP2 (on the right half). a: Photographs were taken 4 dpi for *N. benthamiana*. b: *N. benthamiana* leaves transiently expressing *CaREM1.4* were stained with trypan blue at 5 dpi. Red circles indicate the infiltration areas. B, Immunoblot analysis of the expression of CaREM1.4 protein in *N. benthamiana* leaves. Immunoblotting used an anti-GFP antibody. GFP was used as a negative control; Molecular mass markers in kD are shown on the left. C, Transient *CaREM1.4* overexpression was determined by qRT-PCR. D, qRT-PCR analysis of the expression of immunity-related marker genes, including *NbPR1*, *NbHSR201*, *NbHSR2505*, and *NbPOD* in CaREM1.4 expressed *N. benthamiana* leaves at 24 and 48 hpi, respectively. The relative expression level of marker genes in *N. benthamiana* leaves transiently expressing the empty vector at 24 and 48 hpi were set to “1”. Transcript levels were quantified by qRT-PCR and normalized with *NbEF1α*. Experiments were repeated three times with three independent biological repetitions each time. Bars indicate ± SD. The statistical analyses were performed with Student’s *t* test (***P* < 0.01)


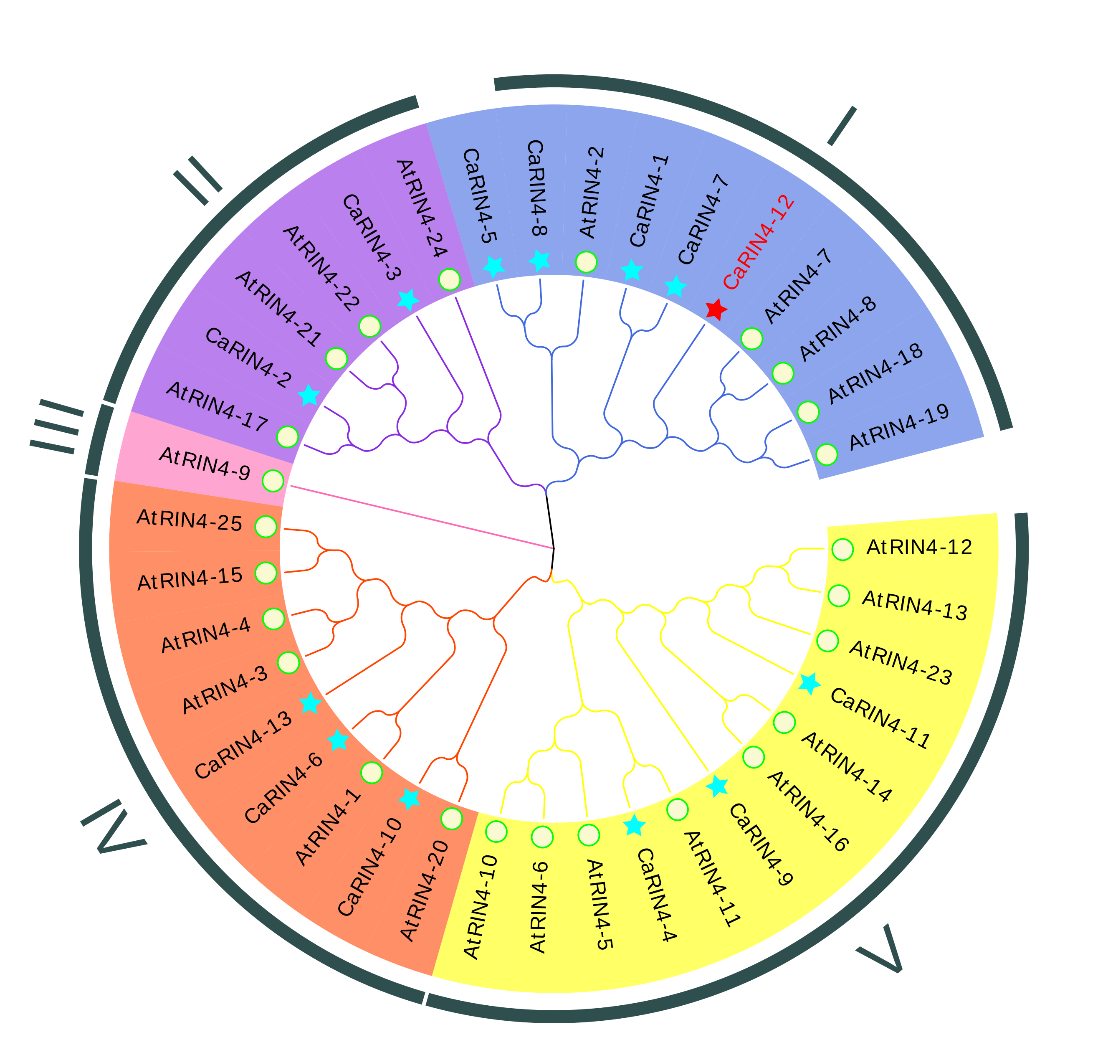


**Supplemental Fig S9.** **Neighbor-joining phylogenetic analysis of RIN4 proteins in *C. annuum*, and *A. thaliana*.** Pepper proteins were indicated in asterisks. The red asterisk indicates the RIN4 protein studied in the manuscript. Different colors were used to highlight different groups, 13 pepper RIN4 proteins were divided into four groups (Groups Ⅰ, Ⅱ, Ⅳ, and Ⅴ).


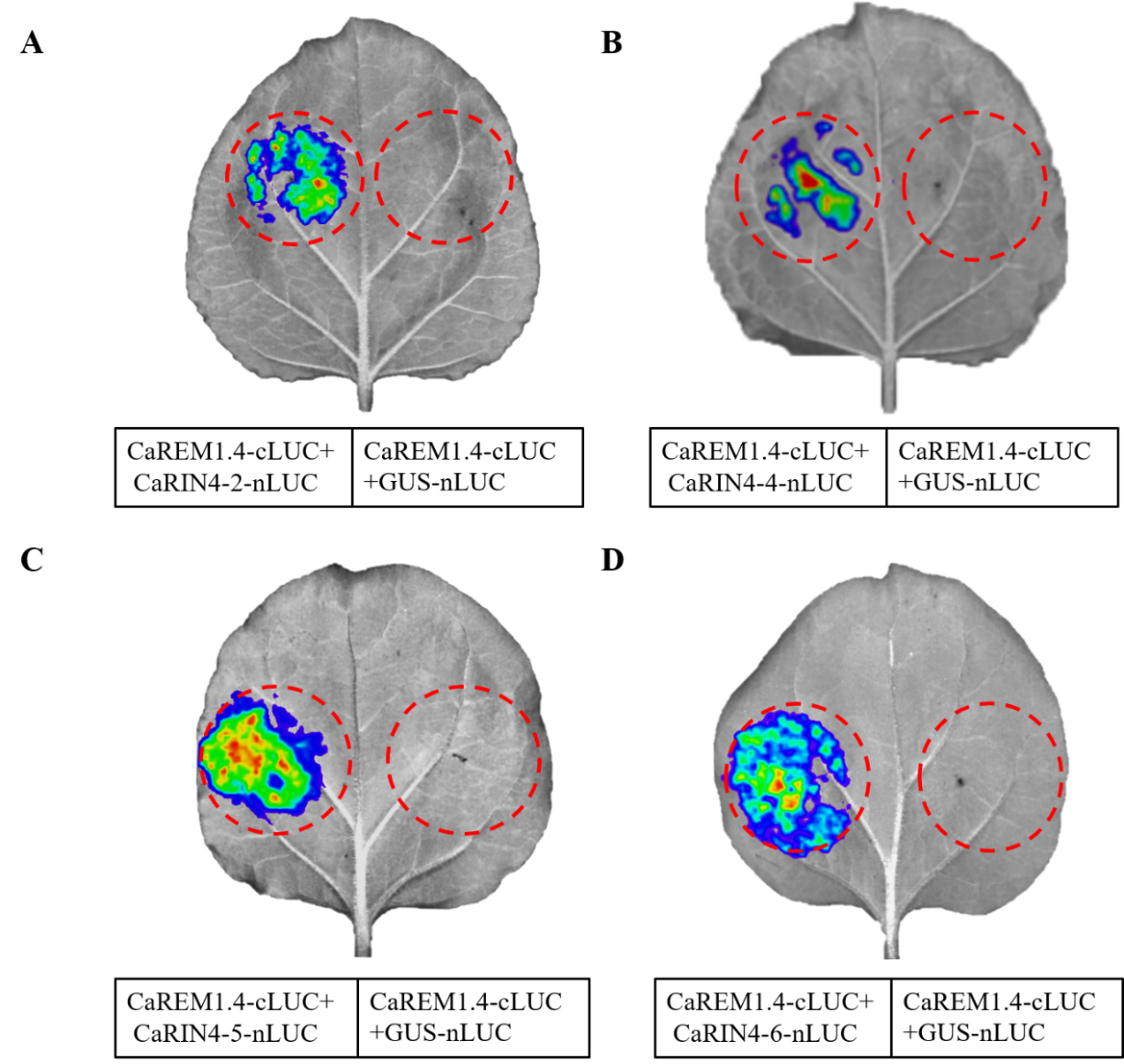


**Supplemental Fig S10.** **CaREM1.4 protein could interact with** **CaRIN4-2 (Groups Ⅱ, A), CaRIN4-4 (Groups Ⅴ, B), CaRIN4-5 (Groups Ⅰ, C), and CaRIN4-6 (Groups Ⅳ, D) by firefly luciferase complementation imaging assay.** The GUS-nLUC was used as negative control.


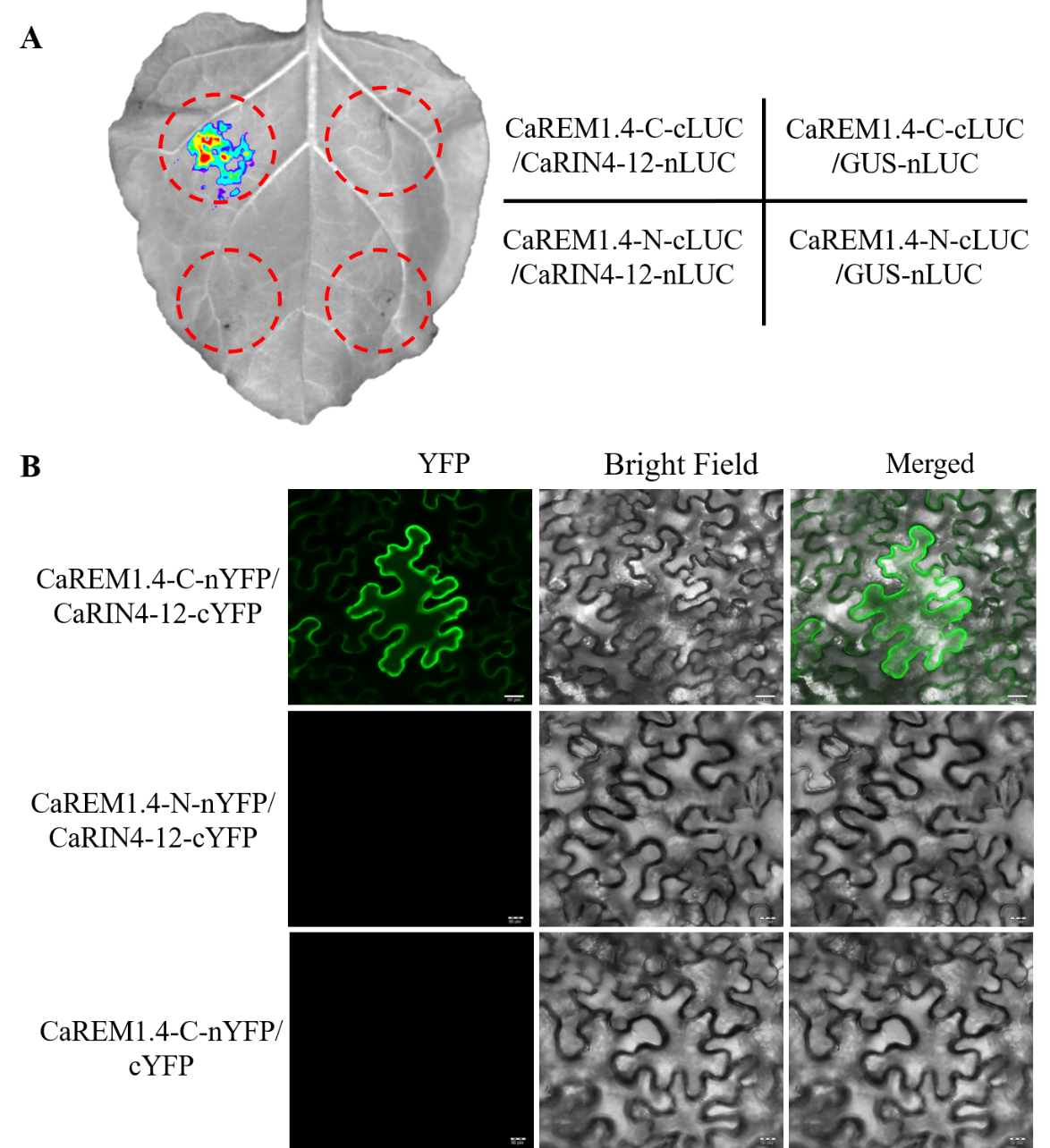


**Supplemental Fig S11. CaRIN4-12 interacts with only the C-terminal domain of CaREM1.4, but not the N-terminal domain.** A, Split-luciferase assays determined the interaction of CaREM1.4-C and CaRIN4-12 in *N. benthamiana* leaves. The GUS-nLUC was used as a negative control. B, C- or N-terminal domain of CaREM1.4 interacted with CaRIN4-12 were verified by BIFC analysis. CaREM1.4-C-nYFP/cYFP was the negative control. YFP fluorescence were taken by confocal microscopy. Bar = 20 μm.


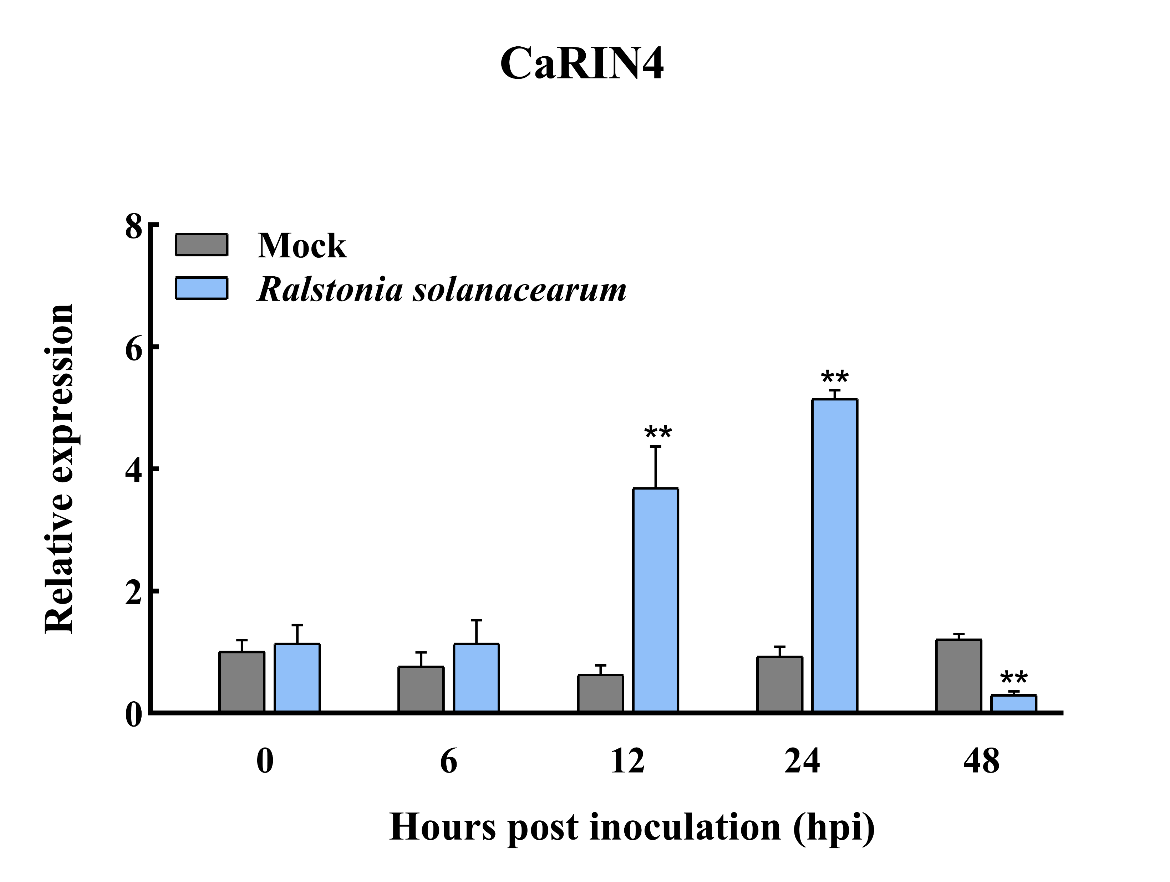


**Supplemental Fig S12.** **The relative transcript levels of *CaRIN4-12* under the treatment of *R. solanacearum*.** The third leaves of pepper plants were infected with the *R. solanacearum* strain Px1 (phylotype I) (OD600 = 0.8) and the mock treatment was injected with 10 mM MgCl_2_. the expression level of *CaRIN4-12* in untreated plants was set to “1”. *CaActin* was used as an internal reference gene. Data represents the means ± SD from three independent values. Asterisks (***P* < 0.01) indicate a significant difference compared with control by Student's *t* test.

**
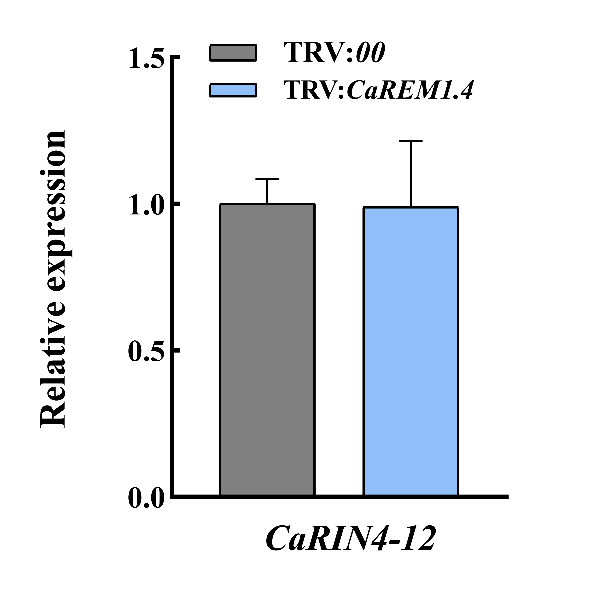
**

**Supplemental Fig S13. The relative transcript levels of the *CaRIN4-12* gene in leaves of TRV:*CaREM1.4* and TRV:*00* plants.** Transcript levels were quantified by RT-qPCR and normalized with *CaActin*. Data are shown as means ± SD from three biological replicates. Statistical significance was determined by Student's *t* test.
