# Supplementary material for: CaREM1.4 interacts with CaRIN4 to regulate Ralstonia solanacearum tolerance by triggering cell death in pepper
Source: Hortic Res. 2023 Mar 28;10(5):uhad053. doi: 10.1093/hr/uhad053 (PMC10199716; doi:10.1093/hr/uhad053)
Supplement: Web_Material_uhad053 [file web_material_uhad053.zip › Supplemental Table S2.doc]

**Table S2.** **Identification of consensus sequence of CaREMs using MEME and the functional sites in motifs by Eukaryotic Linear Motif resource programs (ELM).** The motif numbers are the same to those described in **Supplemental Figure S3.**

| **Motif Number** | **Motif found in proteins** | **Motif size (aa)** | **Motif E-value** range | **Best possible match (amino acid LOGO)** | **Functional sites in Motifs** | | |
| --- | --- | --- | --- | --- | --- | --- | --- |
|  |  |  |  |  | **Positions** | **Cell Compartment** | **ELM Description** |
| Motif 1 | Found in all genes | 41 | 6.19E-36 to 3.21E-19 |   IQAWENLKKAKAEAELKKLEEKLEKKRAEAAEKMKNKIARI | 13-19  15-23  20-28  21-28 | PML body, nucleus | Inverted version of SUMOylation motif recognized for modification by SUMO-1 |
| Motif 2 | Found in all but CaREM5.1 CaREM5.2 CaREM3.2 | 41 | 4.51E-37  to  5.98E-20 |   HKKAEEKRAVAEAKRGEELLKVEEKAAKIRATGQAPKKFLC | 14-22 | nucleus, cytosol | MAPK interacting molecules (e.g. MAPKKs, substrates, phosphatases) carry docking motif that help to regulate specific interaction in the MAPK cascade. |
| Motif 3 | Found in all but CaREM1.5 | 24 | 8.19E-11  to  1.78E-11 |   ESKIKAWEEAEKSKIENKYKREEA | 19-24 | cytosol, internal side of plasma membrane | The C-terminal class 3 PDZ-binding motif is classically represented by a pattern such as (DE)X(VIL) |
| Motif 4 | CaREM5.1CaREM5.3CaREM5.2CaREM6.3CaREM6.2CaREM6.1CaREM6.4 | 26 | 5.69E-31  to  9.98E-20 |   RSVSRRDMGTZMTPIGSQEPSRTGTP | 10-17  14-21  18-25 | cytosol, nucleus | GSK3 phosphorylation recognition site |
| Motif 5 | CaREM5.1CaREM5.3CaREM5.2CaREM6.3CaREM6.2CaREM6.1CaREM6.4 | 26 | 5.71E-29  to  2.29E-19 |   YNSGRPLPSKWDDAEKWIVSPVSGQG | 17-23 | cytosol, nucleus | Proline-Directed Kinase (e.g. MAPK) phosphorylation site in higher eukaryotes |

* Motif 1 represents of Remorin-C domain. * Motif 2 represents of Remorin-N domain. – unknown function.

** ELM. Kumar M, Gouw M, Michael S, Sámano-Sánchez H, Pancsa R, Glavina J, Diakogianni A, Valverde JA, Bukirova D, Čalyševa J, Palopoli N, Davey NE, Chemes LB, Gibson TJ. ELM-the eukaryotic linear motif resource in 2020. Nucleic Acids Res. 2020 Jan 8;48(D1):D296-D306. doi: 10.1093/nar/gkz1030.

*** [http://elm.eu.org](http://elm.eu.org/).

**** https://meme-suite.org/meme/
